# Label-free characterization of single extracellular vesicles using two-photon fluorescence lifetime imaging microscopy of NAD(P)H

**DOI:** 10.1038/s41598-020-80813-0

**Published:** 2021-02-08

**Authors:** Janet E. Sorrells, Elisabeth M. Martin, Edita Aksamitiene, Prabuddha Mukherjee, Aneesh Alex, Eric J. Chaney, Marina Marjanovic, Stephen A. Boppart

**Affiliations:** 1 Beckman Institute for Advanced Science and Technology, University of Illinois at Urbana-Champaign, Urbana, IL 61801 USA; 2 Department of Bioengineering, University of Illinois at Urbana-Champaign, Urbana, IL 61801 USA; 3 GSK Center for Optical Molecular Imaging, University of Illinois at Urbana-Champaign, Urbana, IL 61801 USA; 4 Department of Electrical and Computer Engineering, University of Illinois at Urbana-Champaign, Urbana, IL 61801 USA; 5 Cancer Center at Illinois, Urbana, IL 61801 USA; 6 Carle Illinois College of Medicine, University of Illinois at Urbana-Champaign, Urbana, IL 61801 USA

**Keywords:** Multiphoton microscopy, Biophotonics

## Abstract

The heterogeneous nature of extracellular vesicles (EVs) creates the need for single EV characterization techniques. However, many common biochemical and functional EV analysis techniques lack single EV resolution. Two-photon fluorescence lifetime imaging microscopy (FLIM) is widely used to functionally characterize the reduced form of nicotinamide adenine dinucleotide and nicotinamide adenine dinucleotide phosphate (NAD(P)H) in cells and tissues. Here, we demonstrate that FLIM can also be used to image and characterize NAD(P)H in single isolated EVs. EVs were isolated using standard differential ultracentrifugation techniques from multiple cell lines and imaged using a custom two-photon FLIM system. The presented data show that the NAD(P)H fluorescence lifetimes in isolated cell-derived EVs follow a wide Gaussian distribution, indicating the presence of a range of different protein-bound and free NAD(P)H species. EV NAD(P)H fluorescence lifetime distribution has a larger standard deviation than that of cells and a significantly different fluorescence lifetime distribution than the nuclei, mitochondria, and cytosol of cells. Additionally, changes in the metabolic conditions of cells were reflected in changes in the mean fluorescence lifetime of NAD(P)H in the produced EVs. These data suggest that FLIM of NAD(P)H could be a valuable tool for EV research.

## Introduction

Extracellular vesicles (EVs) are cell-derived membrane-encased particles that contain a wide variety of biomolecules such as proteins, lipids, metabolites, and nucleic acids^1–4^. EVs have recently become a topic of intense investigation due to interest in their function in cell-to-cell communication, applications in therapeutics, and potential as biomarkers of disease^1–3,5–8^. With increasing interest in EVs, there is a growing need to develop new methods of characterization^1–4^. The size distribution and morphology of EVs are commonly characterized using dynamic light scattering (DLS)^9^, nanoparticle tracking analysis (NTA)^10^, electron microscopy^5,11^, and atomic force microscopy (AFM)^12^, but these techniques lack biochemical specificity. Characterization of EV molecular cargo has been achieved using techniques such as Raman spectroscopy^13–15^, Western blotting^16^, mass spectrometry^17,18^, and nucleic acid sequencing^19^. While these techniques provide a broad biochemical profile of bulk EV populations, they lack the ability to analyze the characteristics of individual EVs and to quantify and assess the heterogeneity of EV samples.

Recent progress has been made using optics to characterize single EVs. For example, Raman spectroscopy has been used in conjunction with optical tweezers for single particle trapping and characterization^20^. Additionally, advancements in flow cytometry allowing imaging of smaller particles has recently been applied for single EV characterization^21^. However, flow cytometry requires fluorescence tagging which is time consuming and primarily targets EV surface proteins. There are also concerns about the validation and specificity of fluorescence tags, which causes uncertainty in EV flow cytometry data^1^. Furthermore, both Raman spectroscopy and flow cytometry only report on the biochemical makeup of the EVs, and do not provide information on their intrinsic enzymatic activity, biological functionality, or spatial distribution, which are all critical parameters in EV analysis.

Thus, there exists a need for a non-invasive, single EV characterization method that provides insight on biochemical properties. Recent work has shown promising results with nonlinear optical microscopy, which achieves label-free high spatial resolution (< 500 nm), biochemical specificity, and functional reduction–oxidation (redox) ratio information for single EVs^22,23^. This optical characterization of both isolated and in vivo EVs has been achieved with simultaneous label-free autofluorescence multiharmonic (SLAM) microscopy^22–24^.

Label-free multiphoton characterization of EVs using SLAM microscopy has focused on examining the relative autofluorescence intensity of three essential metabolic cofactors: reduced nicotinamide adenine dinucleotide (NADH), reduced nicotinamide adenine dinucleotide phosphate (NADPH), and flavin adenine dinucleotide (FAD)^22,23^. NADH is a key cofactor in glycolysis, the TCA cycle, and the electron transport chain, while the phosphorylated form, NADPH, plays a central role in biosynthetic pathways and antioxidant defense mechanisms. Collectively, the autofluorescence of NADH and NADPH is combined and referred to as “NAD(P)H” since their excitation and emission spectra are almost identical^25^. Along with NADH, FAD is also essential for the TCA cycle and electron transport chain in the mitochondria. The relative autofluorescence intensities of NAD(P)H and FAD, or the redox ratio, is often used to characterize and quantify the metabolic function of cells and tissues^26,27^. Regarding the redox ratio of EVs, NAD(P)H specifically has drawn interest since a higher NAD(P)H autofluorescence intensity in EVs was correlated with cancer status^22,23,28^. This finding warrants further investigation of the ability to characterize NAD(P)H content of EVs in order to evaluate its potential as a biomarker for disease and its role EV biogenesis and function.

NAD(P)H autofluorescence intensity does not provide complete information on its state or concentration. It has been shown that protein-bound NAD(P)H has a larger quantum yield than free NAD(P)H by approximately tenfold; thus autofluorescence intensity is not directly proportional to NAD(P)H concentration^29,30^. Fortunately, fluorescence lifetime imaging microscopy (FLIM) can be used to better understand the state of NAD(P)H within samples since protein-bound and free NAD(P)H exhibit different characteristic fluorescence lifetimes^31^. Fluorescence lifetime (τ) is the average time between excitation and emission of a fluorescent photon. FLIM is an imaging technique that measures the spatial distribution of fluorescence lifetimes within a sample^32^. In practice, with a sufficient number of photons collected, the fluorescence decay takes the shape of an exponential decay with time constant τ. A detailed explanation of fluorescence lifetime calculation and characterization can be found in Supplementary Note 1. The fluorescence lifetime of fluorophores depends on factors that affect the nanoscale potential energy distribution of electrons such as pH, temperature, and molecular interactions^29,31–33^. The fluorescence lifetime of NAD(P)H in cells and tissues has been widely studied^26,27,34–38^, and it has been found that when bound to a protein, NAD(P)H exhibits a longer fluorescence lifetime of approximately 1.5–6 ns, whereas free NAD(P)H exhibits a characteristically shorter fluorescence lifetime of approximately 0.3–0.8 ns^25,31^.

Another question arising from autofluorescence imaging of NAD(P)H intensity alone is that NADH and NADPH cannot be distinguished. While free NADH and NADPH in a buffer solution cannot be separated using FLIM, protein-bound NADH has a fluorescence lifetime of approximately 1–3 ns and protein-bound NADPH has a fluorescence lifetime of approximately 2–6 ns^25,30,33^. In addition, the fluorescence lifetime of NADH differs slightly when it is bound to different enzymes such as malate dehydrogenase (MDH) and lactate dehydrogenase (LDH), which both also differ from the fluorescence lifetime of NADPH bound to glucose-6-phosphate dehydrogenase (G6PDH)^33^. Thus, FLIM has the potential to probe the state of NADH and NADPH in EVs and provide label-free functional characterization of single EVs based on the relative protein-bound fraction of the two cofactors.

To assess this potential, EVs derived from diverse cell lines were imaged using two-photon FLIM in this proof-of-concept study to quantify their NAD(P)H fluorescence lifetime repeatability and heterogeneity, examine their functional characteristics, and better understand the relationship between EVs and their parent cells. The results show that two-photon FLIM of NAD(P)H in isolated EVs is possible, repeatable, and useful. Noninvasive, label-free optical metabolic imaging using FLIM of NAD(P)H opens up many new possibilities to better study and characterize EVs in the future.

## Results

### Fluorescence lifetime analysis of single EVs

**Figure 1:**
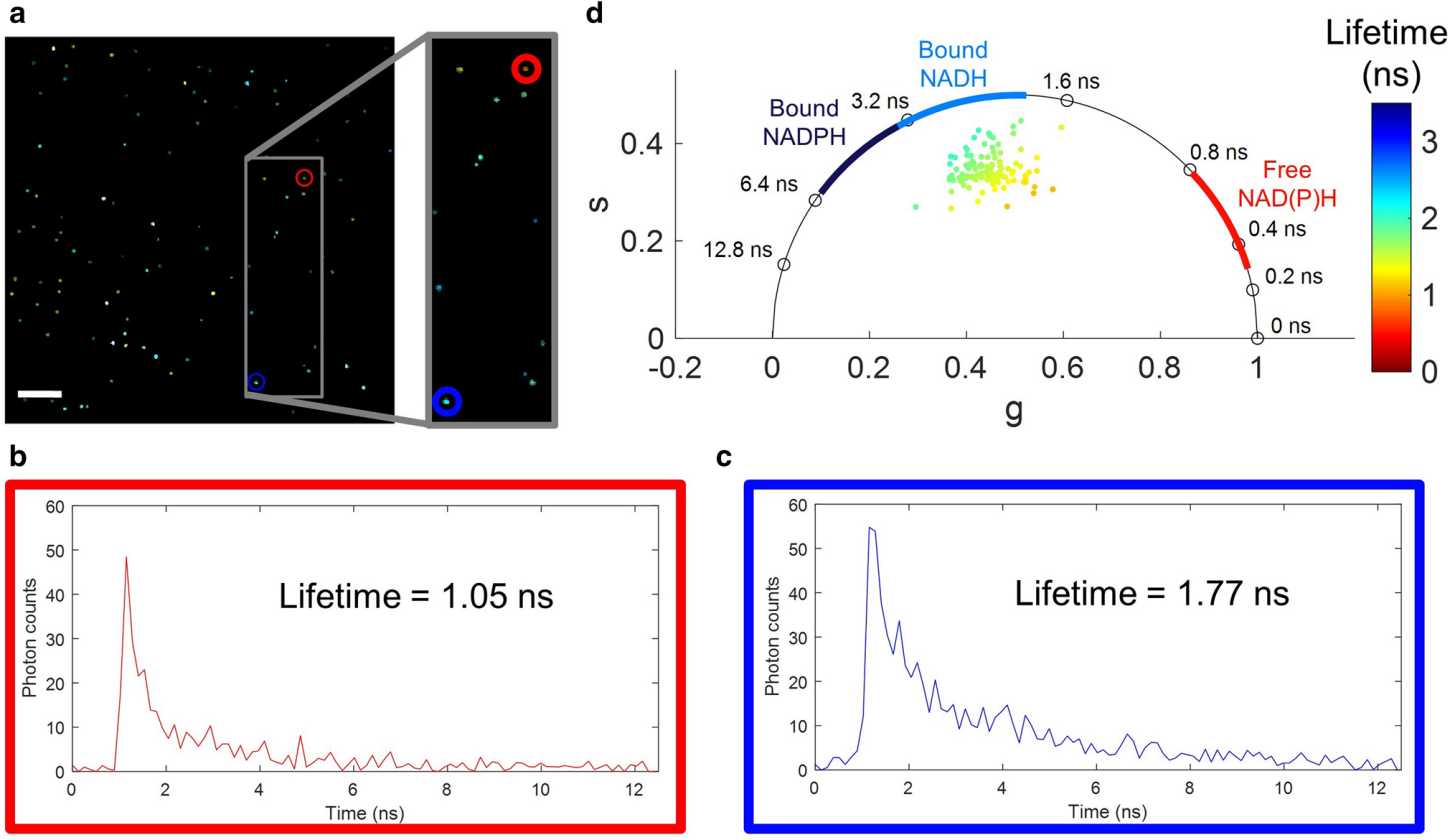
Heterogeneity of fluorescence lifetimes in extracellular vesicles (EVs). (**a**) Fluorescence lifetime-weighted intensity image of EVs derived from MDA-MB-231 cells. The brightness of a pixel corresponds to intensity and the color corresponds to fluorescence lifetime in nanoseconds, using the same fluorescence lifetime scale as in panel (**d**). (**b**) Example fluorescence decay curve of a shorter fluorescence lifetime corresponding to the EV in the red circle in panel (**a**). (**c**) Example fluorescence decay curve of a longer fluorescence lifetime corresponding to the EV in the blue circle in panel (**a**). (**d**) Phasor plot of the frequency components of the exponential decays. Each point on the plot represents a single EV from the image in (**a**). The color of the point represents the fluorescence lifetime of the EV in nanoseconds. Approximate fluorescence lifetime values of protein-bound and free NAD(P)H are labeled based on previous studies^25,33^. Scale bar represents 20 μm.

EVs were isolated from cell conditioned media using differential ultracentrifugation, embedded in a 0.2% (w/vol) agarose gel, and imaged using a previously described custom laser scanning multiphoton setup with FLIM capabilities^34^. The mean fluorescence lifetime, *τ*, of each EV was calculated using the phasor approach for FLIM analysis (Supplementary Fig. S1, Supplementary Note 1), which decomposed the sum of multiple exponential decays into a two-component basis, from which one mean fluorescence lifetime, *τ*, was determined^39^. Phasor analysis is common for FLIM data of NAD(P)H and is ideal for analyzing fluorescence lifetime profiles without making assumptions about the fluorescence lifetime distributions present^30,39^. A single 180 × 180 µm^2^ field-of-view (FOV) fluorescence lifetime-weighted image of EVs derived from MDA-MB-231 cells (Fig. 1a) shows dozens of EVs with fluorescence lifetimes between 0.8 and 2.4 ns. To demonstrate the heterogeneous nature of individual EVs, two example fluorescence decays, one short and one long, are shown in Fig. 1b,c. In Fig. 1d, each EV is represented as a single point in a phasor plot, where the different frequency components *s* and *g* represent a sum of exponential decays. Approximate values of protein-bound NADH and NADPH, and free NAD(P)H are labelled according to previous studies^25,33^.

**Figure 2:**
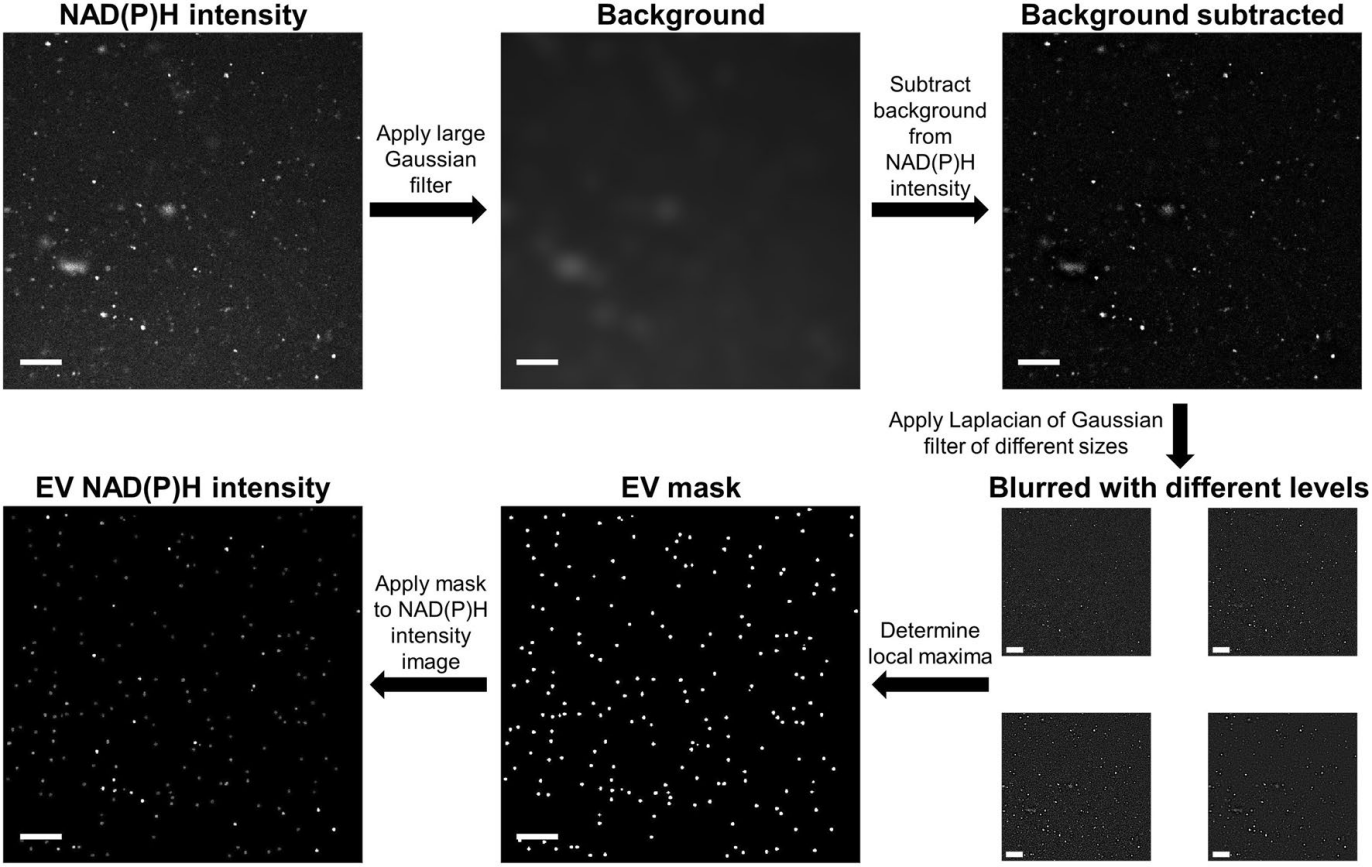
Schematic of extracellular vesicle (EV) segmentation using a blob detection algorithm. First, a large Gaussian filter was applied and then subtracted from the original intensity image to adjust for nonuniformity in the background. Next, Laplacian of Gaussian filters of different sizes were applied to the image to highlight local maxima of varying sizes. A 3D local maxima filter was applied to a stack of images filtered at different levels to create a mask for EVs of different sizes and a threshold was applied to only segment maxima that exceed the background noise. Finally, this binary mask was multiplied by the original intensity image to display the intensity of each segmented EV. Scale bars represent 20 μm.

Single EV segmentation, which enables plotting each EV as a single point on the phasor plot in Fig. 1d, is achieved using a custom blob detection algorithm applied to raw NAD(P)H intensity images. Automated EV detection using this algorithm helps to eliminate the human error associated with manual segmentation. Blob detection is a commonly used feature detection algorithm to detect round objects of different sizes^40^. The image processing procedure for creating an EV segmentation mask is shown in Fig. 2. All image processing was performed using Matlab 2018 (Mathworks, Natick, MA). After the EV segmentation mask was created, the photon count histograms of each pixel within a segmented EV were binned into one representative decay per EV to increase signal, such as those shown in Fig. 1b,c.

A negative control sample of four FOVs from naïve serum-free media that lacked contact with any cells showed only four particles (Supplementary Fig. S2, Supplementary Note 2), likely small contaminants introduced during the isolation and embedded procedures. This is significantly fewer than the hundreds of EVs found in samples isolated from cell conditioned media.

### Assessment of repeatability between measurements

**Figure 3:**
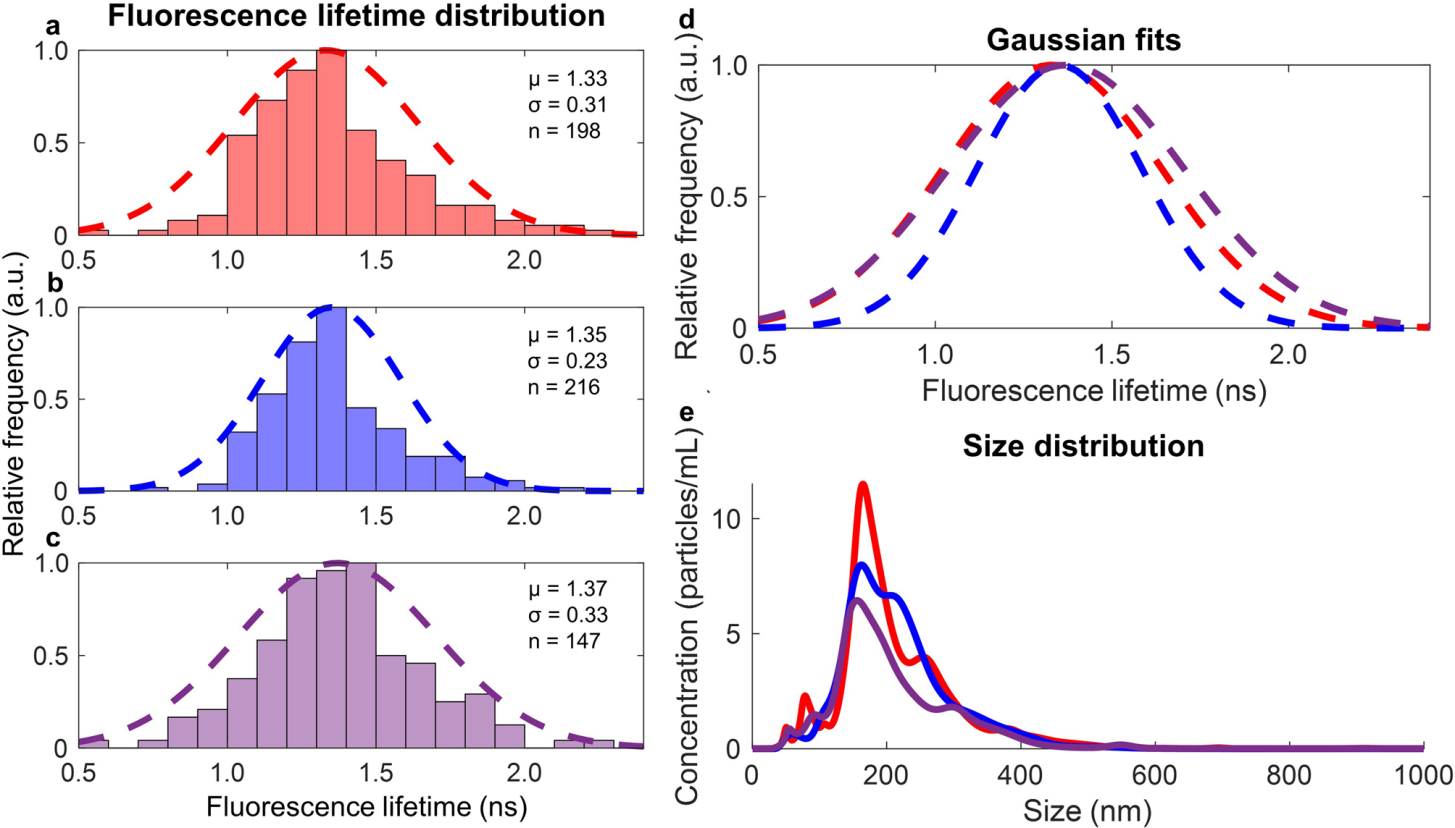
FLIM measurements from three different extracellular vesicle (EV) isolations under similar conditions. (**a**–**c**) Histograms of the relative number of EVs by fluorescence lifetime and a Gaussian fit (*dashed line*). (**d**) Gaussian fits compared with a Student’s t-test; the three samples are not significantly different with *P* > 0.05. (**e**) Nanoparticle tracking analysis (NTA) measurements of concentration and size of isolated EVs for the three isolations. Concentration is calculated to correspond to EVs per mL of cell culture fluid in T175 cell culture flasks with 30 mL of media per flask. First replicate is indicated in red, second in blue, and third in purple.

The repeatability of FLIM measurements was examined using EVs collected from three different sets of MDA-MB-231 cells. The fluorescence lifetime distributions were fit to Gaussian curves and quantified by their mean ± standard deviations: 1.33 ± 0.31 ns (Fig. 3a), 1.35 ± 0.23 ns (Fig. 3b), and 1.37 ± 0.33 ns (Fig. 3c). Replicates were found to be not significantly different from each other by Student’s t-test (*P* > 0.05). An overlay of the normalized Gaussian fit curves is shown in Fig. 3d. The concentrations and size distributions for the replicates were also measured with NTA (Fig. 3e).

### Comparison of isolated EVs and their parent cells

**Figure 4:**
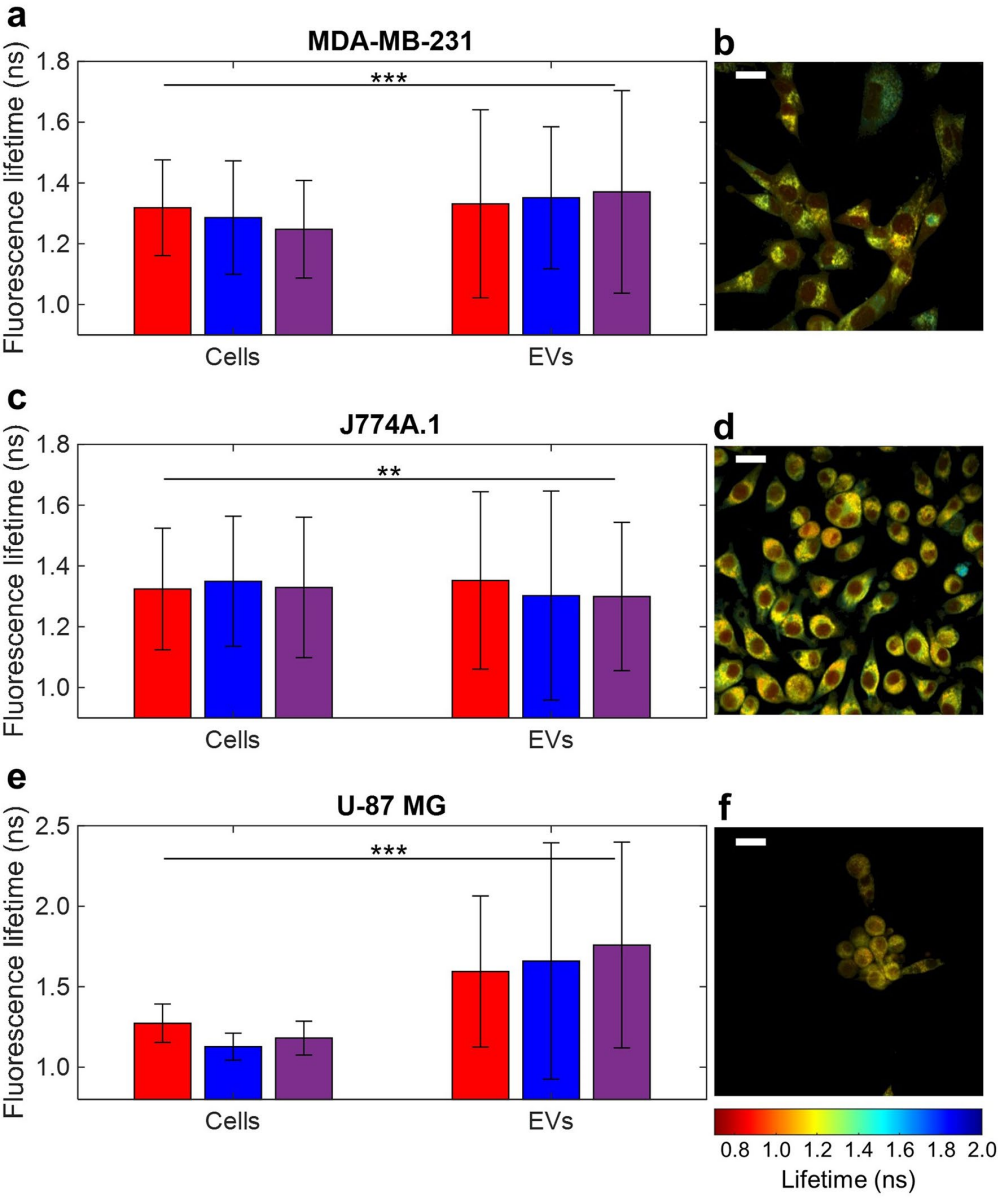
Fluorescence lifetime values of extracellular vesicles (EVs) and their parent cells for three cell lines: (**a**,**b**) MDA-MB-231 human epithelial breast cancer, (**c**,**d**) J774A.1 mouse macrophage, and (**e**,**f**) U-87 MG human glioblastoma. Mean and standard deviation of fluorescence lifetime values are indicated in the bar charts for three different replicates of each cell line, with each replicate indicated by color (replicate 1: *red*, replicate 2: *blue*, replicate 3: *purple*), in (**a**,**c**,**e**). Each replicate consisted of 4 fields-of-view (FOVs) for cells and 16 FOVs for EVs. One example FOV of each cell line is given as well: (**b**) MDA-MB-231, (**d**) J774A.1, and (**f**) U-87 MG. **Indicates *P* < 0.01, *** indicates *P* < 0.001. First replicate is indicated in red, second in blue, and third in purple. Scale bars represent 20 μm. The total number of vesicles (n) for each experimental group pooled together is as follows: MDA-MB-231 n = 561, J774A.1 n = 968, U-87 MG n = 178.

The metabolic states of three different parent cell lines (MDA-MB-231 human epithelial breast cancer, J774A.1 mouse monocyte/macrophage, and U-87 MG human glioblastoma) and the EVs they produced were compared using FLIM of NAD(P)H. As shown in Fig. 4a,c,e, the distribution of fluorescence lifetimes present in EVs is longer and has a larger standard deviation than the distribution of fluorescence lifetimes present in pixels from cells imaged using the same parameters for all three cases. The fluorescence lifetime distributions of parent cells and their corresponding EVs were found to be significantly different across all three cell lines using a Student’s t-test (*P* < 0.01) with three replicates, indicated by color, in Fig. 4a,c,e. For reference, representative fluorescence lifetime weighted images of MDA-MB-231 (Fig. 4b), J774A.1 (Fig. 4d), and U-87 MG (Fig. 4f) cells are also shown.

**Figure 5:**
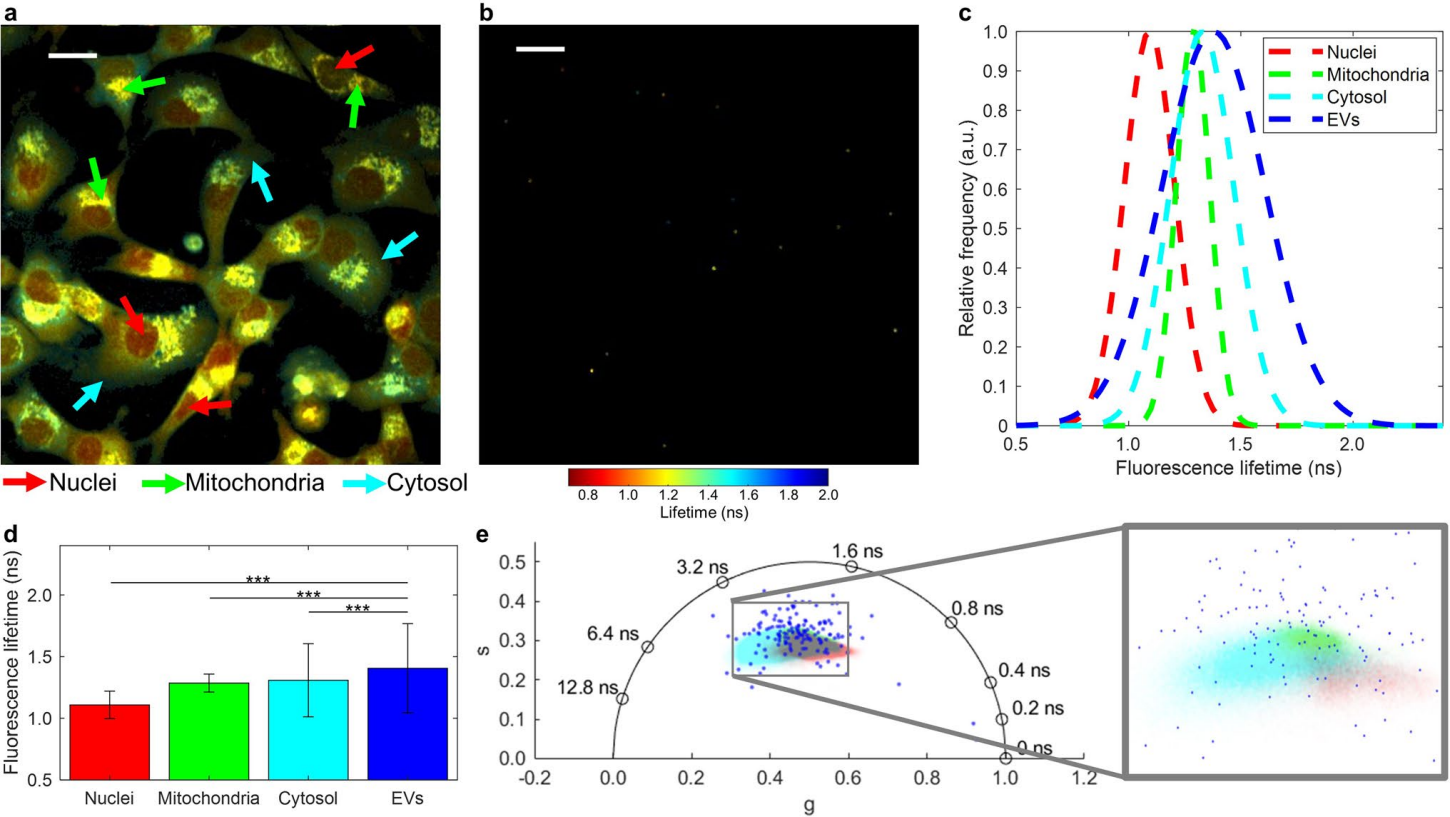
FLIM signature of extracellular vesicles (EVs) and the cellular components of their parent cells. (**a**) Fluorescence lifetime-weighted image of MDA-MB-231 cells with corresponding arrows for nuclei (*red*), mitochondria (*green*), and cytosol (*cyan*). (**b**) Fluorescence lifetime-weighted image of EVs. Color bar for FLIM images given in ns. (**c**) Fluorescence lifetime Gaussian fits of major cellular components and EVs. (**d**) Bar chart showing mean and standard deviation of fluorescence lifetime distributions. *** indicates *P* < 0.001. (**e**) Phasor plot showing the phasor components of EVs (*blue*) and the cellular nuclei (*red*), mitochondria (*green*), and cytosol (*cyan*). Scale bars represent 20 μm.

In order to provide a more comprehensive analysis and fully utilize the capabilities of FLIM, the fluorescence lifetime distribution of pixels within one FOV of MDA-MB-231 cells was separated by manual segmentation into three major cellular components: nuclei, mitochondria, and cytosol. Examples of these cellular components are indicated with arrows in Fig. 5a, which shows a fluorescence lifetime weighted image of the MDA-MB-231 cells. For visual comparison, a fluorescence lifetime weighted image of the EVs derived from the cells is presented in Fig. 5b. Gaussian distributions (mean ± standard deviation) were fit to the fluorescence lifetime histograms of each cellular component; the nuclei (1.10 ± 0.11 ns), mitochondria (1.29 ± 0.07 ns), and cytosol (1.30 ± 0.30 ns) all had a shorter, more narrow fluorescence lifetime distributions than the EVs (1.41 ± 0.36 ns), and were all significantly different (*P* < 0.001) than the EV fluorescence lifetimes, as shown in Fig. 5c-d. The fluorescence lifetime profiles of each cellular component and of the EVs were also plotted on a phasor scatterplot (Fig. 5e) to allow for visual comparison.

### Measuring functional metabolic changes in EVs using FLIM

**Figure 6:**
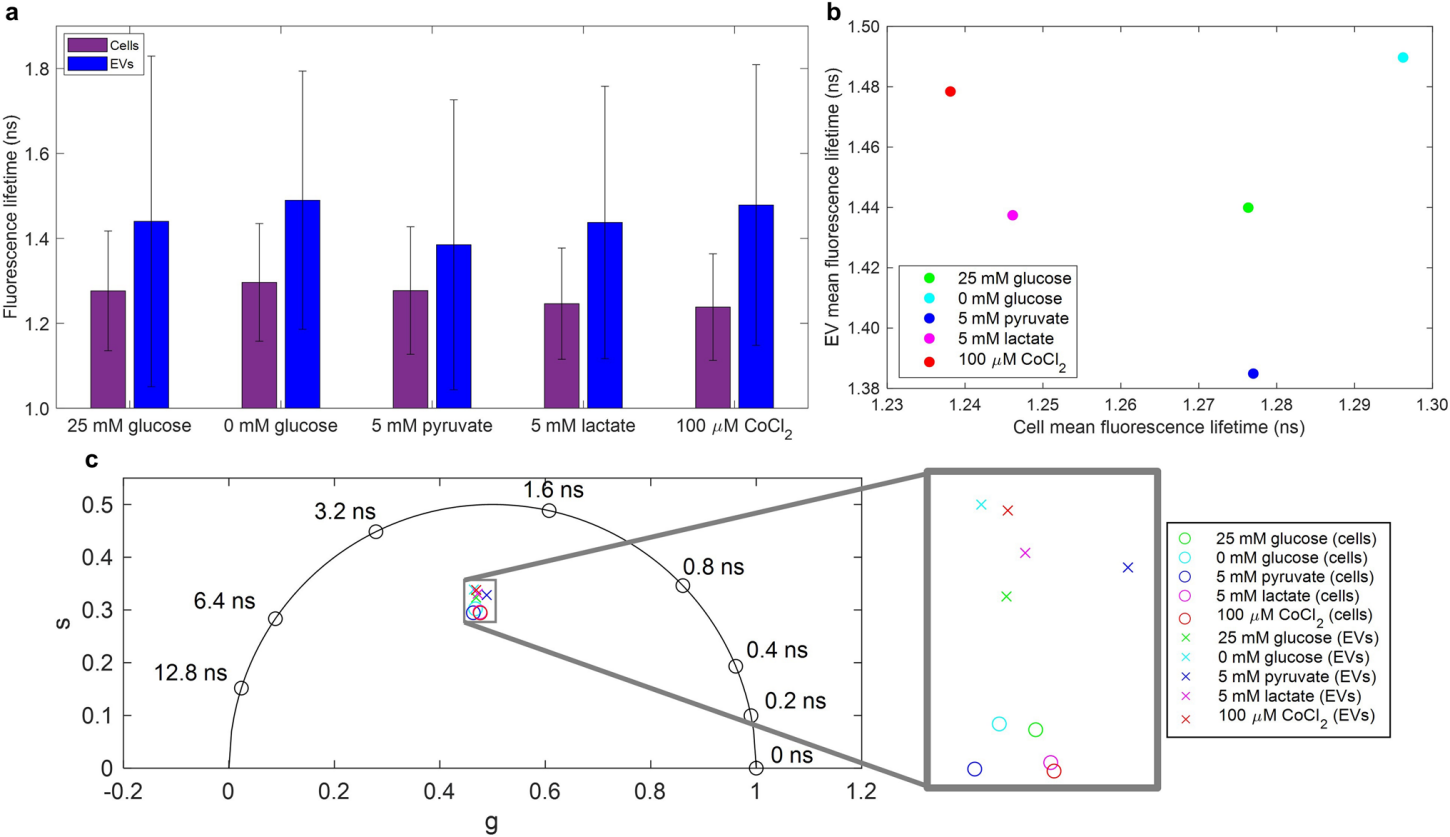
The effect of cellular metabolic conditions on the fluorescence lifetime of cells and cell-derived extracellular vesicles (EVs). (**a**) Mean and standard deviation of fluorescence lifetime values of cells (*purple*) and EVs (*blue*) are indicated in the bar charts for the five experimental conditions; each condition contains pooled data from three independent replications. (**b**) Scatterplot of EV mean fluorescence lifetime vs. cell mean fluorescence lifetime for the five experimental groups: 25 mM glucose (*green*), 0 mM glucose (*cyan*), 5 mM pyruvate (*blue*), 5 mM lactate (*magenta*), and 100 μM CoCl_2_ (*red*). (**c**) Phasor components of cell (*o markers*) and EV (*x* *markers*) mean fluorescence lifetimes. Experimental group is indicated by color as described above. Data from three replicates of each experimental condition is pooled together, and each replicate consisted of 4 fields-of-view (FOVs) for cells and 16 FOVs for EVs. The total number of vesicles (n) for each experimental group pooled together is as follows: 25 mM glucose n = 629, 0 mM glucose n = 2003, 5 mM pyruvate n = 864, 5 mM lactate n = 1668, 100 µM CoCl_2_ n = 1363.

To investigate the effect of altered parent cell metabolism on EV NAD(P)H fluorescence lifetime, MDA-MB-231 cells were incubated for 48 h in the presence or absence of glucose, sodium lactate, or sodium pyruvate. An additional subset of cells was kept under chemically induced hypoxia, which has previously been shown to decrease the bound ratio and bound lifetime of NAD(P)H in cells^41^. Data from three independent replicates was pooled together for each group. Consistent with the previously presented data, all treatment groups show that the EV fluorescence lifetime distribution is higher and wider than that of cells (Fig. 6a). Data can be found in tabular format in Supplementary Table S1, and is represented with histograms and Gaussian fits in Supplementary Figs. S3, S4. One way analysis of variance (ANOVA) analysis indicates that among the treatment groups, there are differences in the mean fluorescence lifetime values for both EVs (*P* < 0.001) and cells (*P* < 0.001). However, a multiple comparisons analysis of EV fluorescence lifetime shows that three of the ten group pairings are not significantly different (*P* > 0.05): 25 mM glucose vs. 5 mM lactate (*P* = 0.9999), 25 mM glucose vs. 100 μM CoCl_2_ (*P* = 0.1244), and 0 mM glucose vs. 100 μM CoCl_2_ (*P* = 0.8641). All of the ten pairings are significantly different (*P* < 0.05) for the cell fluorescence lifetime data. A table summarizing the multiple comparisons results is presented in Supplementary Table S2; it is important to note that multiple *P* values between groups fall between 0.05 and 0.01.

There was no linear relationship between EV and cell mean fluorescence lifetime for the collected data across the five different metabolic conditions (Fig. 6b; R^2^ = 0.00139, *P* = 0.953). Figure 6c provides a more comprehensive examination of the different fluorescence lifetime distributions using phasor analysis. The phasor plot shows some separation between experimental groups that is not visible by examining lifetime alone, such as between the EVs originating from cells with 25 mM glucose and 5 mM lactate.

## Discussion

In this proof-of-concept study, we show for the first time that FLIM can be used to image and characterize NAD(P)H autofluorescence originating from single EVs. Though there are many different ways to characterize EVs, FLIM of NAD(P)H offers the advantages of single EV analysis, functional metabolic information, spatial information, and straightforward comparison with live samples such as cells. Single EV analysis allows for characterization of the heterogeneity of the sample, something that is lost in techniques like Western blotting, protein concentration assays, mass spectrometry, and nucleic acid sequencing. Unlike other single particle analysis techniques like TEM, NTA, or flow cytometry, FLIM of NAD(P)H provides information about endogenous functional and biochemical status.

Single EV analysis (Fig. 1) suggests that cell-derived EVs have a variety of protein-bound and free NAD(P)H species. Single EVs are segmented using an automated blob detection algorithm (Fig. 2). After isolation, EVs are resuspended in PBS with 25 mM trehalose, which has been shown to prevent aggregation^42^, though there is still the possibility that some segmented single EVs may in fact be multiple EVs aggregated together. With the ability to image hundreds of EVs in minutes, the heterogeneity and distribution of EV fluorescence lifetimes can also be characterized and fit to a Gaussian distribution (Fig. 3a–d). The fluorescence lifetimes present in EVs from three separate isolations were not significantly different, which indicates that FLIM of NAD(P)H in EVs is repeatable. However, in order to further investigate this phenomenon, in the future it will be important to characterize the NAD(P)H fluorescence lifetimes from EVs derived from a variety of sources such as saliva, cerebrospinal fluid, urine, and blood plasma or serum.

One advantage of nonlinear optical microscopy techniques, such as two-photon FLIM, is that they can be used for high resolution (< 500 nm) imaging of live cells under the same imaging parameters as their corresponding EVs (Fig. 4). The fluorescence lifetime distributions of the cells and EVs were compared and found to be significantly different for three different cell lines. However, the fluorescence lifetime profiles of the EVs overlap with the cellular fluorescence lifetime profiles, especially in the case of MDA-MB-231 (Fig. 4a) and J774A.1 (Fig. 4c) cells, whereas EVs derived from U-87 MG cells showed a much longer mean fluorescence lifetime than cells and a larger EV fluorescence lifetime standard deviation (Fig. 4e). This suggests that the relationship between cell NAD(P)H and EV NAD(P)H may differ based on the characteristics of the parent cells. It is also important to note that cell images were collected from 20 frames taken over nearly 2 min, the same imaging parameters used for EVs. Since the cells are alive, it is expected that some motion within the cells may have contributed to averaging over time, which could lead to a lower calculated standard deviation of fluorescence lifetime values than is present instantaneously in the cells. Additionally, it is possible that the reduced SNR of some of the EV images contributed to less precise EV NAD(P)H fluorescence lifetime calculations and a higher fluorescence lifetime standard deviation than the cell fluorescence lifetime distribution.

The relationship between EVs and parent cells is investigated further in Fig. 5, by segmenting the fluorescence lifetime of MDA-MB-231 cells into cellular compartments. The NAD(P)H autofluorescence in cells is generally assumed to be primarily from NADH, since there is about 10 times more NAD+/NADH in cells than NADP+/NADPH^29^. This implies that the subpopulation of EVs that overlaps with the cells on the phasor plot (Fig. 5e) likely also has a FLIM signature dominated by NADH. Furthermore, as shown in Fig. 5c,d, although the fluorescence lifetime values of the EVs are significantly different from those of the main cellular components, they have the most overlap with the values associated with the cytosol, rather than the nuclei or mitochondria of live cells. The cytosol also has a broader fluorescence lifetime profile than the mitochondria or nucleus.

Previous proteomic studies that have examined the proteome of parent cells and their resulting EVs suggest that the makeup of EVs is closely related to that of the parent cells; EV content is most similar to the cytosol, and is less likely to be similar in composition to the cell nucleus or mitochondria^2,6,7,43–45^. There are a high number of proteins present in EVs that are involved in cellular metabolism and the anti-oxidant response, some of which use NADH or NADPH as cofactors or substrates^6,46–56^. A non-comprehensive list of NAD(P)H-related proteins that have been found in EVs is presented in Supplementary Table S3. Furthermore, many proteins are enriched in EVs, some of which can have up to 100 × higher concentration in EVs than in cell lysate^8^. Protein enrichment in EVs could lead to more protein-bound NAD(P)H in EVs, which could account for the longer NAD(P)H lifetime observed in EVs compared to cells in Figs. 4, 5, 6. These data provide examples of how the label-free high resolution live-cell imaging capabilities of FLIM can contribute to better understanding not only EVs, but also their relationship with cell biology. Fluorescence lifetime can also be altered by factors other than protein binding that affect the rate of internal rotation of the molecule, such as viscosity and temperature^57^. It is possible that tight confinement of NAD(P)H in EVs may restrict NAD(P)H rotation and increase the fluorescence lifetime, though any contribution from this phenomenon appears to be relatively small.

FLIM of NAD(P)H has been used to characterize and study cancerous cells and tissues due to their altered metabolic function, such as the often observed shift toward cytosolic glycolysis^26,35,36^. Conversely, changes in parent cell conditions, such as hypoxia, have been shown to alter the physical and biochemical properties of the resulting EVs^43,58,59^. Here, we demonstrate that EVs derived from MDA-MB-231 cells under different metabolic conditions (Fig. 6) produce EVs with significant differences in mean fluorescence lifetimes. In the main cellular glucose pathway, glucose is split into two pyruvate molecules, creating cytosolic “free” NADH, and pyruvate is sent into the mitochondria for the TCA cycle (where NADH is created) and electron transport chain (where NADH is bound to Complex I and converted to NAD+). In the cytosol, pyruvate can also be converted to lactate, converting NADH to NAD+, and the backward reaction can happen as well. The electron transport chain, which requires oxygen, can be inhibited by chemical hypoxia, such as that induced by CoCl_2_^41^. Thus, each of the five conditions presented in Fig. 6 will perturb the cellular metabolism in a slightly different way. Various other studies have shown similar shifts in the fluorescence lifetime profile of cells under different metabolic conditions^27,33,60^, yet Sharick et al. also note that the interpretation of these results is not always clear^33^.

Though more studies need to be done to fully understand the causality behind the relationship between parent cell metabolism and EVs, these findings show that the NAD(P)H fluorescence lifetime of EVs is related to the metabolic state of their parent cells, further supporting the usefulness of the FLIM for characterizing EVs. This contributes to the evidence that EVs could be used as biomarkers of disease, since cellular metabolic changes indicative of cancer are present in EVs, as suggested by our previous work using SLAM microscopy^22,23^. Furthermore, it has been shown that tumor-related EVs can reprogram the metabolism of cells that uptake them^61–63^, and that malignant cells create more EVs than normal cells^64^. Many have stressed the importance of understanding the metabolic and functional role of EVs in cancer^4,65,66^, and the presented data indicate that future work examining the fluorescence lifetime of NAD(P)H in EVs could contribute to understanding and quantifying their metastatic potential in liquid biopsy samples. There are also still underlying questions about the packaging of EVs^8^, and future work using time-lapse FLIM could potentially be used to better elucidate the process of EV release. Our data demonstrate how multiphoton microscopy techniques such as FLIM offer label-free possibilities for visualizing spatial information of cells and single EVs. The presented data focused on isolated EVs, but it will be important for future studies to additionally image EV in vivo and in vitro, since the effect of the isolation and embedding procedure on NAD(P)H fluorescence lifetime is unknown.

Recent work used coregistered hyperspectral coherent anti-Stokes Raman (CARS) and multiphoton microscopy to characterize the spatial distribution and biochemical profile of EVs in vivo^28^. Raman microscopy provides useful information about the macromolecular makeup and can report on different types of molecules, unlike FLIM or SLAM. However, it lacks the functional aspect for quantifying cellular metabolic indicators that can be performed with NAD(P)H imaging. Present work using FLIM is limited to EVs that contain NAD(P)H and would require multimodal detection to examine a larger population of EVs via alternative microscopy methods. As more microscopy techniques are developed for EV imaging, we can characterize EVs in their native environments and bypass the issues related to EV isolation protocols, and thus move closer towards filling the immense need for more in vivo experiments and a more comprehensive understanding of EV biology^4^.

The results presented in this study show that FLIM of NAD(P)H has potential as a technique for imaging and functionally characterizing single EVs in order to assess EV heterogeneity, fulfilling a need that has not previously been met^67^. The EVs produced by MDA-MB-231, U-87 MG, and J774A.1 cells are heterogenous and follow a broad Gaussian distribution of fluorescence lifetimes of NAD(P)H that is significantly different than their parent cells. The capabilities of FLIM to compare live cells and their isolated EVs were investigated, showing that in terms of an NAD(P)H FLIM signal, EVs were most similar to the cytosol. This supports previous findings that EVs are similar in composition to cytosol but can be enriched with proteins capable of binding NAD(P)H. Fluorescence lifetime was also used to differentiate between populations of EVs from cells under different metabolic conditions. Like all EV analysis techniques, FLIM of EVs will require standardization before widespread use becomes feasible. FLIM and other label-free microscopy techniques have not yet been explored broadly for EV analysis, and future studies could provide valuable and unique insight into the metabolic and cellular origins of EVs, as well as explore their diagnostic capabilities.

## Materials and methods

### Cell culture

MDA-MB-231 triple-negative human epithelial breast cancer epithelial cells (ATCC HTB-26, Manassas, VA), were primarily used to produce EVs to allow for comparison with previous work with nonlinear optical imaging^22^. Additionally, U-87 MG human glioblastoma (ATCC HTB-14, Manassas, VA), and J774A.1 mouse BALB/c monocyte/macrophage (ATCC TIB-67, Manassas, VA), cell lines were used to diversify the EV samples used for examination of FLIM capabilities. MDA-MB-231 and J774A.1 cells were grown in a complete phenol-free Dulbecco’s Modified Eagles Medium (DMEM, Gibco, Waltham, MA) supplemented with 4 mM L-glutamine, 10% heat-inactivated Fetal Bovine Serum (FBS, GE Healthcare Life Sciences, HyClone Laboratories, Logan, UT), and 1% streptomycin-penicillin antibiotic solution (PSA, Thermo Fisher Scientific, Waltham, MA). U-87 MG cells were grown in a complete phenol-free Minimum Essential Medium alpha (MEM alpha, Gibco, Waltham, MA) supplemented with 10% heat-inactivated FBS (GE Healthcare Life Sciences, HyClone Laboratories, Logan, UT) and 1% PSA (Thermo Fisher Scientific, Waltham, MA). All cells were maintained at 37 °C in a humidified 5% CO_2_ incubator. Cell concentration and viability protocol and measurements for selected experiments are available in Supplementary Note 3 and Supplementary Table S4.

Cells designated for imaging were plated 48 h prior to imaging on poly-d-lysine coated 35 mm glass bottom dishes (MatTek Life Sciences, Ashland, MA; P35GC-0-14-C). Approximately 24 h prior to cell imaging, complete cell media was replaced with the corresponding serum-free media.

### EV isolation by differential ultracentrifugation

A serial differential ultracentrifugation procedure was followed based on previous similar studies and isolation protocols^1,2,4,6,22^. Once the cells reached 70–90% confluency, complete media was removed and cells were rinsed twice with sterile 1 × Phosphate Buffered Saline (PBS, GE Healthcare Life Sciences, HyClone Laboratories, Logan, UT) and incubated with the corresponding serum-free media for 48 h. Media was then collected into 50 mL PCR-clean Protein LoBind Eppendorf tubes (Eppendorf, Hauppauge, NY), and centrifuged at 800×*g* for 10 min to pellet cells, then at 2000×*g* for 30 min to pellet particles larger than EVs, and finally at 12,000×*g* for 60 min to pellet EVs. The choice to image the EVs pelleted at 12,000×*g* is justified in Supplementary Note 4 and Supplementary Table S5. To prevent pellet disintegration and final sample contamination, during each media transfer to new tubes between centrifugation steps, approximately 2.5 mL of media was left above the pellet. All centrifugation steps were performed at 4 °C. The obtained EV pellet was resuspended in 10–20 μL of sterile PBS (twice passed through 50 nm pore size PES syringe filter) per each 10 mL of initial cell media in PCR-clean Protein LoBind Eppendorf tubes (Eppendorf, Hauppauge, NY). As a negative control, the differential ultracentrifugation procedure was performed on naïve serum-free media that had not been incubated with any cells (Supplementary Fig. S2).

### Nanoparticle tracking analysis (NTA) and EV validation

For enumeration and size distribution, EVs were characterized with NTA (Nanosight NS300, Malvern, UK) using the 488 nm laser. From the initial solution of isolated EVs, 10 μL was diluted into 990 μL sterile PBS. Diluted EVs were analyzed with NTA using a constant injection speed and a sterile injection syringe. Six videos of 30 s each were collected per sample. EV number and size distribution were calculated by NTA software version 3.3. Final EV concentrations were calculated based on the number of EVs per the volume of initial cell culture media. TEM and Raman spectroscopy were also used for validation of selected experiments to ensure that the isolated particles resembled EVs (Supplementary Fig. S5, Supplementary Note 5). EV concentration and mean diameter from NTA for all reported experiments are in Supplementary Table S6.

### Metabolic perturbation of MDA-MB-231 cells and EVs

To examine the effect of metabolic perturbations of parent cells on their corresponding EVs, MDA-MB-231 cells were incubated with phenol-free, serum-free, and glutamine-free DMEM with 1% PSA, and either: 25 mM glucose, 0 mM glucose, 0 mM glucose + 5 mM sodium pyruvate (11360070, Gibco, Waltham, MA), 0 mM glucose + 5 mM sodium L-lactate (71781, Sigma-Aldrich, St. Louis, MO), or 25 mM glucose + 100 μM CoCl_2_ (ACROS Organics, Thermo Fisher Scientific, Waltham, MA). The first four conditions alter the energy metabolism of the cells whereas CoCl_2_ induces chemical hypoxia^41^.

After reaching 70–90% confluence, three T175 flasks were designated to each condition. Cell were rinsed twice with sterile 1 × PBS and overlain with the corresponding supplemented serum-free DMEM for their metabolic condition. Cells were returned to the incubator for 48 h and isolated as previously described. On the same day, cells in alternate T175 flasks with DMEM and FBS were designated for cell imaging and were plated (48 h prior to imaging) on poly-d-lysine coated glass bottom dishes (MatTek Life Sciences, Ashland, MA; P35GC-0-14-C), with three dishes assigned to each treatment group. Approximately 24 h prior to cell imaging, complete cell media was removed and replaced with serum-free DMEM supplemented for the corresponding metabolic perturbation.

### Fluorescence lifetime imaging microscopy (FLIM)

To prepare EVs for FLIM imaging, isolated EVs were embedded in a 0.2% (w/vol) agarose gel. The gel was heated to its melting point, cooled to approximately 37 °C, and then mixed with EVs in a 5:1 (vol:vol) ratio. This mixture was immediately plated on an uncoated 35 mm glass bottom dish (MatTek Life Sciences, Ashland, MA; P35G-0-14-C) and set in the refrigerator at 4 °C for several minutes to allow the gel to solidify prior to imaging. Agarose gel does not contribute a significant background autofluorescence (Supplementary Figs. S6, S7) and does not change the NAD(P)H fluorescence lifetime properties of EVs (Supplementary Fig. S8). All samples were imaged within an hour after isolation was completed. This stabilization in gel was important to prevent motion during image acquisition, which required up to 2 min per field-of-view.

EV and cell samples were imaged at room temperature (about 25 °C) using a previously described custom laser scanning multiphoton setup with FLIM capabilities^34^. The glass bottom imaging dishes allow for illumination and detection from below the sample. A tunable femtosecond laser (Discovery Chameleon, Coherent, Santa Clara, CA) was tuned to the excitation wavelength of 750 nm, and two-photon NAD(P)H autofluorescence was collected after a bandpass filter centered at 451 nm (FF01-451/106-25, Semrock, Rochester, NY), consistent with previous studies^34^. Fluorescence lifetime information was collected using a hybrid photon counting photomultiplier detector (PMA-40 mod, Picoquant, Berlin-Adlershof Germany) and a commercial time correlated single photon counting (TCSPC) unit (HydraHarp 400, PicoQuant, Berlin-Adlershof, Germany).

For each pixel within the image, photons were tagged and binned by the time delay between the laser pulse and when the photon arrived at the detector, creating a histogram of photon counts vs. time for each pixel. A time bin size of 8 ps was used for data collection, and data was summed into 128 ps bins for data analysis for all reported experiments. Fluorescence lifetime calculations were performed using phasor analysis with frequency domain deconvolution, as described in Supplementary Note 1. Since the NAD(P)H fluorescence signal from EVs was relatively low, 20 frames were accumulated and summed for each 180 × 180 µm^2^, 512 × 512 pixels field-of-view. To image sufficient EVs for analysis, 16 fields-of-view were acquired per sample, which took around 30 min in total; for cell imaging, 4 fields-of-view were collected per dish.

### Data and statistical analysis

Analysis and visualization of data was performed using Matlab 2018 (Mathworks, Natick, MA). Statistical significance between groups of fluorescence lifetimes was determined using a two-sided Student’s t-test, one-way ANOVA, and/or multiple comparisons test using Matlab 2018 (Mathworks, Natick, MA), as indicated. A *P* value of < 0.05 was considered significant.

## Supplementary Information

Supplementary Information

## Data Availability

The data and code that support these results are available from the corresponding author upon reasonable request and through collaborative investigations.
