# Supplementary material for: Label-free characterization of single extracellular vesicles using two-photon fluorescence lifetime imaging microscopy of NAD(P)H
Source: Sci Rep. 2021 Feb 8;11:3308. doi: 10.1038/s41598-020-80813-0 (PMC7870923; doi:10.1038/s41598-020-80813-0)
Supplement: Supplementary file 1 — Supplementary Information [file 41598_2020_80813_MOESM1_ESM.pdf]

Label-free characterization of single extracellular vesicles using two-photon fluorescence lifetime imaging microscopy of NAD(P)H

Janet E. Sorrells[1,2], Elisabeth M. Martin[2], Edita Aksamitiene[1,3], Prabuddha Mukherjee[1,3], Aneesh Alex[1,3], Eric J. Chaney[1], Marina Marjanovic[1,2,3], Stephen A. Boppart[1,2,3,4,5,6,*]

[1]*Beckman Institute for Advanced Science and Technology, University of Illinois at Urbana-Champaign, Urbana, IL 61801, USA*

[2]*Department of Bioengineering, University of Illinois at Urbana-Champaign, Urbana, IL 61801, USA*

[3]*GSK Center for Optical Molecular Imaging, University of Illinois at Urbana-Champaign, Urbana, IL 61801, USA*

[4]*Department of Electrical and Computer Engineering, University of Illinois at Urbana-Champaign, Urbana, IL 61801, USA*

[5]*Cancer Center at Illinois, Urbana, IL 61801, USA*

[6]*Carle Illinois College of Medicine, University of Illinois at Urbana-Champaign, Urbana, IL 61801, USA*

[*]*Corresponding author, boppart@illinois.edu*

Supplementary Note 1: Phasor analysis of fluorescence lifetimes[1,2,3,4]

The fluorescence lifetime, or average time between fluorophore excitation and fluorescent photon emission, is given by equation (1).

$$\tau = \frac{1}{k_r + k_{nr}} \qquad (1)$$

Here, k_r is the rate of radiative (fluorescent) decay, and k_{nr} is the rate of non-radiative (quenching) decay. The number of fluorophore molecules present in the excited state (n) at a given time (t) can then be characterized with the following differential equations in equations (2a,b).

$$n'(t) = -(k_r + k_{nr})n(t) \qquad (2a)$$

$$n(t) = n_0 e^{-t(k_r + k_{nr})} = n_0 e^{-(t/\tau)} \qquad (2b)$$

Thus, the time between excitation and fluorescence emission follows an exponential decay probability function for a given fluorophore at constant conditions. This means that the probability (p) of a photon being emitted after a given amount of time (t) follows equation (3).

$$p(t) = \alpha e^{-(t/\tau)} \qquad (3)$$

The α variable is a constant for normalization. In a non-hypothetical environment, molecular interactions and nanoscale differences in variables such as temperature and pH cause differences in fluorescence lifetime. When a group of fluorophores in different conditions is probed, the corresponding probability function of time between excitation and emission will be the sum of many decaying exponentials, as shown in equation (4).

$$s(t) = \sum \alpha e^{-(t/\tau)} \qquad (4)$$

The α variable now represents the relative intensity of each species present.

Another factor at play in fluorescence lifetime measurements is the detector time response function, $d(t)$, since the detector does not provide an instantaneous response. The repetition rate of the laser, f, can also be represented in radians as ω_o, or $2\pi f$. Each pulse of the laser will cause the emission of photons that follow the shape of $s(t)$ and are detected as the convolution of $d(t)$ and $s(t)$, as shown in equation (5) below, where the measured fluorescence is given by $f(t)$.

$$f(t) = d(t) * s(t) * comb(\frac{2\pi t}{\omega_o}) \qquad (5)$$

From here, the challenge is to characterize the unknown variable $s(t)$ with measured $f(t)$ and knowledge of $d(t)$ and as ω_o. Equation (5) represented the time domain, but this equation can easily be converted into the frequency domain in equation (6) and rearranged in equation (7).

$$F(\omega_o) = D(\omega_o)S(\omega_o) \qquad (6)$$

$$S(\omega_o) = F(\omega_o)/D(\omega_o) \tag{7}$$

Next, the frequency domain variables $F(\omega_o)$ and $D(\omega_o)$ are calculated from their known time domain counterparts and split into a normalized real (g) and imaginary (s) basis as shown in equations (8a-c) and equations (9a-c).

$$F(\omega_o) = f(t)\cos(\omega_o t) + jf(t)\sin(\omega_o t) \tag{8a}$$

$$g_f = \frac{f(t)\cos(\omega_o t)}{\int f(t)} \tag{8b}$$

$$s_f = \frac{f(t)\sin(\omega_o t)}{\int f(t)} \tag{8c}$$

$$D(\omega_o) = d(t)\cos(\omega_o t) + jd(t)\sin(\omega_o t) \tag{9a}$$

$$g_d = \frac{d(t)\cos(\omega_o t)}{\int d(t)} \tag{9b}$$

$$s_d = \frac{d(t)\sin(\omega_o t)}{\int d(t)} \tag{9c}$$

These basis values are then used to define $S(\omega_o)$, and decompose $S(\omega_o)$ into its own normalized real (g) and imaginary (s) basis, as shown in equation (10a-c).

$$S(\omega_o) = \frac{g_f + js_f}{g_d + js_d} = g_s + js_s \tag{10a}$$

$$g_s = \frac{g_f g_d + s_f s_d}{(g_d)^2 + (s_d)^2} \tag{10b}$$

$$s_s = \frac{-g_f s_d + s_f g_d}{(g_d)^2 + (s_d)^2} \tag{10c}$$

These two basis components should fall within the unit square (but due to noise in experimental measurements, they occasionally do not) as represented in **Supplementary Fig. S1**. Any sum of exponential decays can be fully described by these two basis components, and the mean fluorescence lifetime of the sum of decays is calculated with equation (11).

$$\tau = \frac{s_l}{\omega_o g_l} \tag{11}$$

Supplementary Figure S1 shows five different fluorescence lifetime profiles. In red, τ_1 represents a pure fluorescence lifetime around 0.6 ns; τ_2 in light blue is a pure fluorescence around 3 ns; τ_3 in dark blue is a pure fluorescence lifetime around 4 ns; τ_4 and τ_5 both represent mixtures with mean fluorescence lifetimes of 1.6 ns. Mixtures of multiple fluorescence lifetimes are represented at a location between the fluorescence lifetimes present, weighted by relative intensity, α. For example, the τ_4 location represents a mixture of τ_1 and τ_2, where the ratio of the intensity of fluorophores with fluorescence lifetime of τ_1 to fluorescence lifetime τ_2 is a_1:a_2.

The usefulness of phasor analysis of fluorescence lifetimes is highlighted by the ability to distinguish τ_4 and τ_5 as having different fluorescence lifetime components, despite having the same mean fluorescence lifetime.

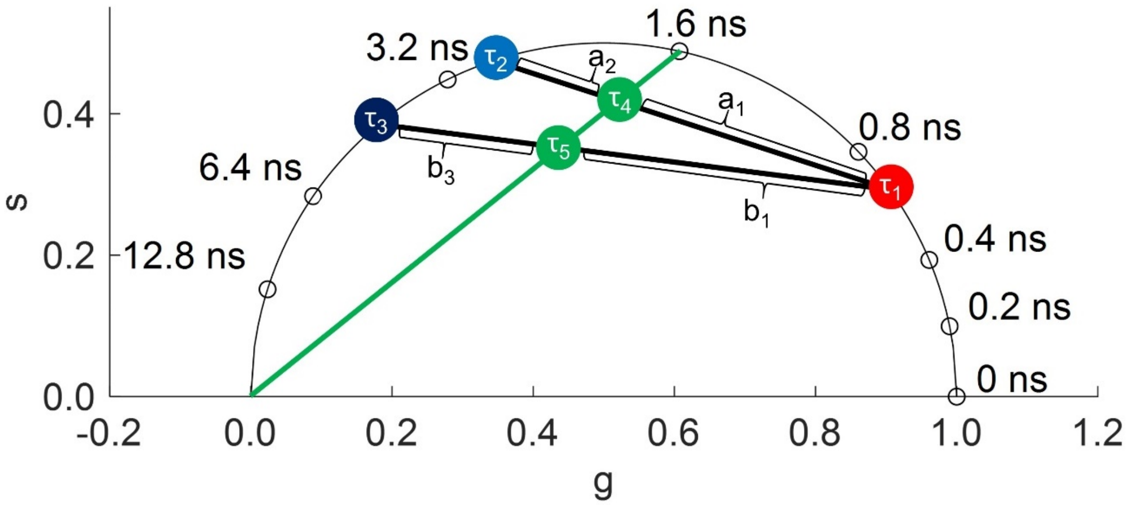

Supplementary Figure S1. Example phasor plot with 5 fluorescence lifetime profiles represented. Measured fluorescence decays are decomposed into a two component basis (g and s) that can be visualized on this plot.

Supplementary Note 2: Negative control sample

To verify that the particles being imaged with FLIM were cell-derived EVs, the differential centrifugation, sample preparation, and image processing procedures were performed on naïve serum-free media that was not incubated with any cells. As can be seen in **Supplementary Fig. S2**, only four particles were found in the automated blob detection segmentation, and of those only one contained a fluorescence lifetime profile falling on the phasor plot. Likely, these particles are not EVs containing NAD(P)H, but are some other type of particulate or contaminant that is scattering light, and not fluorescing. Similar results were obtained using EV diluent (sterile PBS that was previously passed through 50 nm PES syringe filter twice). These data provide evidence that the particles imaged throughout the main text are cell-derived.

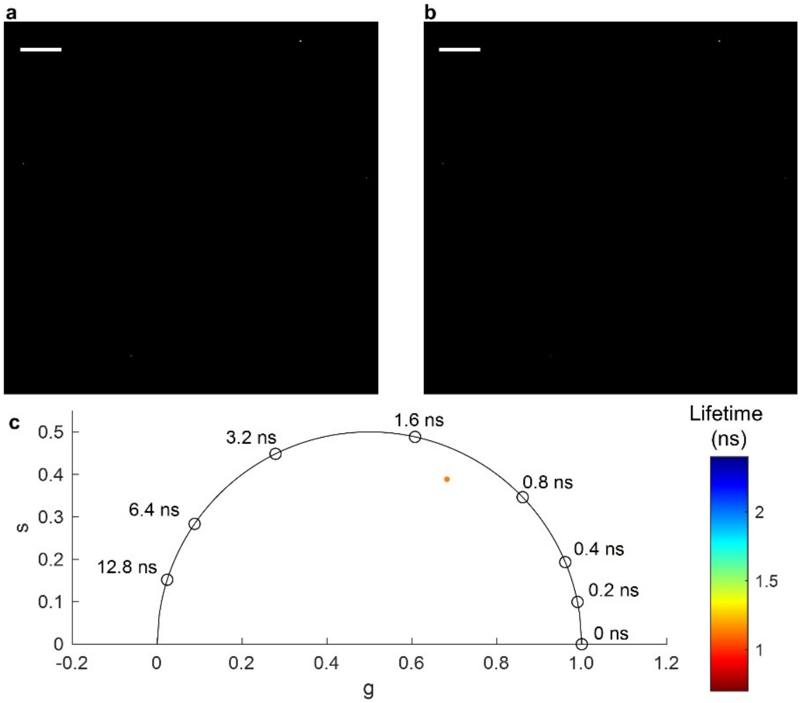

Supplementary Figure S2. **FLIM of particles isolated from unused media.** (a) EV NAD(P)H intensity after segmentation; comparable with the EV NAD(P)H intensity in **Fig. 2** (main text). (b) Fluorescence lifetime-weighted intensity image; comparable with **Fig. 1a** (main text). Fluorescence lifetime values in nanoseconds are given by the colorbar in (c). (c) Phasor plot of fluorescence lifetime profiles present in unused media; comparable with **Fig. 1d** (main text). Fluorescence lifetime values given in nanoseconds. Scale bar represents 40 μm.

Supplementary Table S1. Cell and EV fluorescence lifetime distributions under different metabolic conditions.

Group	Cell fluorescence lifetime mean ± standard deviation (ns)	EV fluorescence lifetime mean ± standard deviation (ns)
25 mM glucose	1.276 ± 0.141	1.440 ± 0.389
0 mM glucose	1.296 ± 0.139	1.490 ± 0.304
5 mM pyruvate	1.291 ± 0.150	1.385 ± 0.341
5 mM lactate	1.246 ± 0.131	1.437 ± 0.321
100 µM $CoCl_2$	1.238 ± 0.125	1.478 ± 0.330

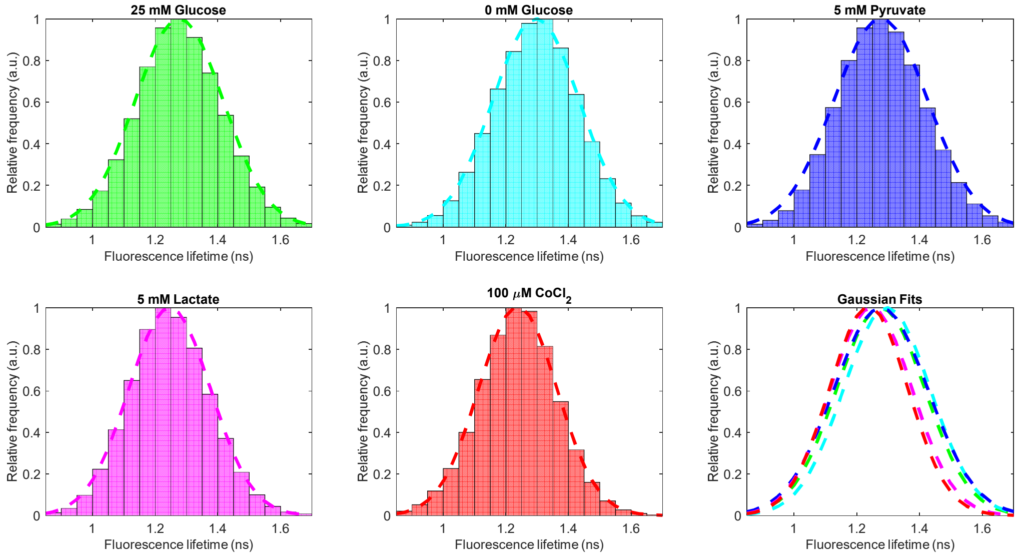

Supplementary Figure S3. Fluorescence lifetime distribution in MDA-MB-231 cells in different metabolic conditions. Histograms show the relative number of pixels by fluorescence lifetime and a Gaussian fit (*dashed line*) for cells in DMEM under 5 conditions: 25 mM Glucose, 0 mM Glucose, 0 mM Glucose + 5 mM pyruvate, 0 mM Glucose + 5 mM lactate, and 25 mM Glucose + 100 µM $CoCl_2$. Lower right panel shows an overlay of the Gaussian fits for each group.

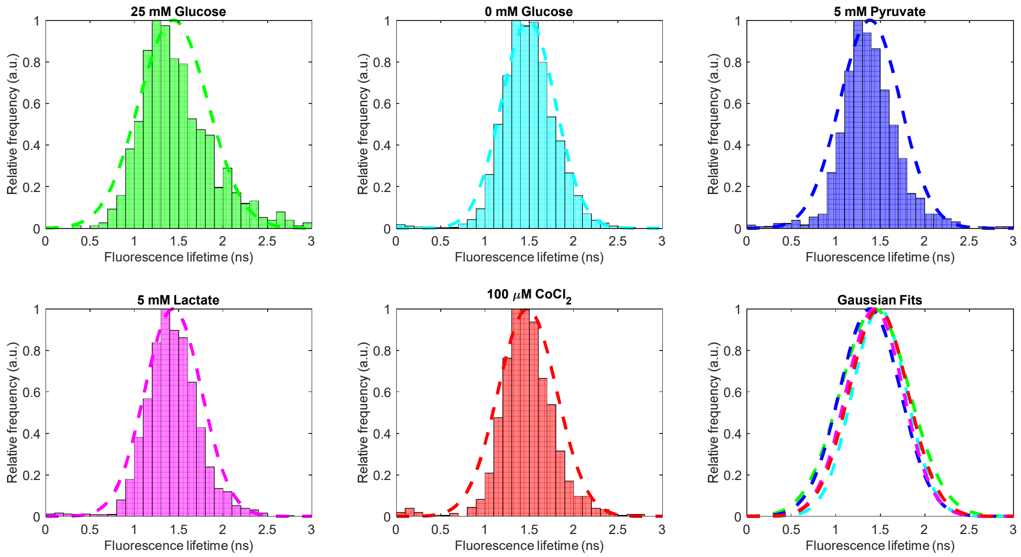

Supplementary Figure S4. Fluorescence lifetime distribution of EVs derived from MDA-MB-231 cells in different metabolic conditions. Histograms show the relative number of pixels by fluorescence lifetime and a Gaussian fit (*dashed line*) for EVs derived from cells in DMEM under 5 conditions: 25 mM Glucose, 0 mM Glucose, 0 mM Glucose + 5 mM pyruvate, 0 mM Glucose + 5 mM lactate, and 25 mM Glucose + 100 µM $CoCl_2$. Lower right panel shows an overlay of the Gaussian fits for each group.

Supplementary Table S2. Multiple comparisons results for EV and cell fluorescence lifetime distributions under different metabolic conditions.

Group 1	Group 2	*P* value from EV fluorescence lifetime	*P* value from cell fluorescence lifetime
25 mM glucose	0 mM glucose	0.0114	0.0000
25 mM glucose	5 mM pyruvate	0.0154	0.0274
25 mM glucose	5 mM lactate	0.9999	0.0000
25 mM glucose	100 µM $CoCl_2$	0.1244	0.0000
0 mM glucose	5 mM pyruvate	0.0000	0.0000
0 mM glucose	5 mM lactate	0.0000	0.0000
0 mM glucose	100 µM $CoCl_2$	0.8641	0.0000
5 mM pyruvate	5 mM lactate	0.0012	0.0000
5 mM pyruvate	100 µM $CoCl_2$	0.0000	0.0000
5 mM lactate	100 µM $CoCl_2$	0.0054	0.0000

Supplementary Table S3. NAD(P)H-related proteins found in EVs.

Gene name	NADH or NADPH	Primary cellular location	References
GAPDH	NADH	cytosol	5-13
LDHA	NADH	cytosol	6, 7, 10-12
LDHB	NADH	cytosol	6, 8, 10
LDHC	NADH	cytosol	10
PHGDH	NADH	cytosol	6
MDH1	NADH	cytosol	5, 6, 11
HPGD	NADH	cytosol	9
CYB5R1	NADH	cytosol	9
ADH1A	NADH	plasma membrane	8, 11
ADH1B	NADH	plasma membrane	8, 11
PDHA	NADH	mitochondria	6, 10
PDHB	NADH	mitochondria	6
ALDH 7A1	NADH	mitochondria	6
Q9NX14	NADH	mitochondria	10
ALDH6A1	NADH	mitochondria	8
G6PD	NADPH	cytosol	6, 7, 8
PGD	NADPH	cytosol	6*, 7-9
GRHPR	NADPH	cytosol	6
AKR1A1	NADPH	cytosol	9, 10
IDH1	NADPH	cytosol	6-9
IDH2	NADPH	cytosol	6, 8
BLVRB	NADPH	cytosol	6, 7, 9
ALDH1L1	NADPH	cytosol	11
GLUD1	NADPH	mitochondria	6, 8
DHRS2	NADPH	mitochondria	11
AKR1D1	NADPH	nucleus	11
CBR1	NADPH	nucleus	7, 9
POR	NADPH	nucleus	11
NQO1	NADPH, NADH	cytosol	6-8
CD38	NADPH, NADH	plasma membrane	14*, 15+
CD157	NADPH, NADH	plasma membrane	15+

Gene names, NADH or NADPH affiliation, primary cellular location, and references for various proteins found in EVs. All cited studies used mass spectrometry for protein identification unless indicated with * for Western Blot or + for flow cytometry.

Supplementary Note 3: Cell concentration and viability

Prior to experiments, cell confluency was determined visually to be about 70% on the day cells were switched to serum-free media. On the day of EV isolation, cell viability was measured with Vi-CELL Cell Viability Analyzer (Beckman Coulter, Horsham, PA). Immediately after serum-free media was removed for EV isolation, cells were removed from culture flasks and re-diluted in complete media. Consistent with ATCC protocols, 0.25% trypsin in HBSS was used to lift MDA-MB-231 and U87 MG cells from their flasks, followed by adding complete media to the trypsin and cell solution, centrifugation of that mixture at 125×g for 5 minutes to pellet cells, and finally resuspending the cells in complete media. A cell scraper was used to remove J774A.1 cells from their flasks. Once cells were resuspended in complete media, they were diluted in a 1:2 ratio with phenol red-free 1x TrypLE Select Enzyme (Gibco, Waltham, MA) and set in an incubator for 5 minutes to prevent clumps of cells. After 5 minutes, the cells were run through the Vi-CELL analyzer using 50 images to calculate cell concentration and viability using a software algorithm for Trypan Blue exclusion assay. Cell concentration, experimental conditions, viability, and concentration are given in **Supplementary Table S4**.

Supplementary Table S4. **Cell line, conditions, viability, and concentration for reported experiments.**

Cell Line	Replicate #	Experimental Conditions	Viability	Concentration (cells/mL)
MDA-MB-231	1	(**Figure 3a**, **Figure 4a,b**)	91.3%	0.56×10^6
MDA-MB-231	2	(**Figure 3b**, **Figure 4a,b**)	94.8%	0.63×10^6
MDA-MB-231	3	(**Figure 3c**, **Figure 4a,b**)	94.0%	0.50×10^6
	Mean ± SE		93.4 ± 1.1%	$0.56 \pm 0.04 \times 10^6$
J774A.1	1	(**Figure 4c,d**)	83.1%	0.46×10^6
J774A.1	2	(**Figure 4c,d**)	75.5%	0.65×10^6
J774A.1	3	(**Figure 4c,d**)	79.5%	0.67×10^6
	Mean ± SE		79.4 ± 2.2%	$0.59 \pm 0.07 \times 10^6$
U87 MG	1	(**Figure 4e,f**)	95.6%	0.17×10^6
U87 MG	2	(**Figure 4e,f**)	95.0%	0.17×10^6
U87 MG	3	(**Figure 4e,f**)	95.4%	0.18×10^6
	Mean ± SE		95.3 ± 0.2%	$0.17 \pm 0.00 \times 10^6$
MDA-MB-231*	1	25 mM Glucose (**Figure 6**)	97.8%	1.47×10^6
MDA-MB-231*	2	25 mM Glucose (**Figure 6**)	98.2%	1.68×10^6
MDA-MB-231*	3	25 mM Glucose (**Figure 6**)	97.5%	1.74×10^6
	Mean ± SE		97.8 ± 1.6%	$1.63 \pm 0.08 \times 10^6$
MDA-MB-231*	1	0 mM Glucose (**Figure 6**)	91.9%	1.40×10^6
MDA-MB-231*	2	0 mM Glucose (**Figure 6**)	92.0%	1.40×10^6
MDA-MB-231*	3	0 mM Glucose (**Figure 6**)	93.6%	1.31×10^6
	Mean ± SE		92.5 ± 0.6%	$0.37 \pm 0.03 \times 10^6$
MDA-MB-231*	1	5 mM pyruvate (**Figure 6**)	91.8%	0.96×10^6
MDA-MB-231*	2	5 mM pyruvate (**Figure 6**)	91.4%	1.08×10^6
MDA-MB-231*	3	5 mM pyruvate (**Figure 6**)	92.8%	1.08×10^6
	Mean ± SE		92.0 ± 0.4%	$1.04 \pm 0.04 \times 10^6$
MDA-MB-231*	1	5 mM lactate (**Figure 6**)	89.7%	1.10×10^6
MDA-MB-231*	2	5 mM lactate (**Figure 6**)	90.0%	1.02×10^6
MDA-MB-231*	3	5 mM lactate (**Figure 6**)	90.3%	1.18×10^6
	Mean ± SE		90.0 ± 0.2%	$1.10 \pm 0.05 \times 10^6$
MDA-MB-231*	1	100 μM $CoCl_2$ (**Figure 6**)	95.9%	1.48×10^6
MDA-MB-231*	2	100 μM $CoCl_2$ (**Figure 6**)	97.0%	1.50×10^6
MDA-MB-231*	3	100 μM $CoCl_2$ (**Figure 6**)	97.6%	1.47×10^6
	Mean ± SE		96.8 ± 0.5%	$1.48 \pm 0.01 \times 10^6$

Viability and concentration data was measured with Vi-CELL Cell Viability Analyzer. Concentration is calculated to correspond to cells/mL of cell culture fluid in T175 cell culture flasks with 30 mL of media per flask. Mean and standard error of the mean for each set of three isolations is calculated. * indicates that cells pelleted after the 800×g ultracentrifugation step were added into adherent cells for concentration and viability; otherwise only adherent cells were measured.

Supplementary Note 4: FLIM of large vs. small EVs

Serum-free cell culture fluid was sequentially centrifuged at 800×g for 10 minutes to pellet cells, then at 2,000×g for 30 minutes to pellet particles larger than 1000 nm, at 12,000×g for 60 minutes to pellet large EVs (LEVs), and at 100,000×g for 60 minutes to pellet small EVs (SEVs) to compare the capability of FLIM to measure LEVs and SEVs. **Supplementary Table S5** provides information on the size, concentration, and number of EVs detected in FLIM for large and small EVs. Concentration is calculated to correspond to mL of initial cell culture fluid used. Mean diameter and concentration were determined with NTA as described in the main text. Equivalent samples of SEVs and LEVs showed more LEVs than SEVs in FLIM, but more SEVs than LEVs in NTA, which means that a higher proportion of LEVs are detected with NAD(P)H FLIM.

Volume is cubically related to diameter, so as EVs get smaller, they contain much less material. For example, this could cause an EV of diameter 50 nm to appear 8 time less bright than an EV of diameter 100 nm if both EVs had the same concentration of NAD(P)H. Furthermore, it is possible that small EVs contain less NAD(P)H or less bound NAD(P)H, which could further account for fewer of them being visible. These two factors are suspected to be the cause for fewer SEVs being detected. Future work will need to better characterize the NAD(P)H content of SEVs.

Supplemental Table S5. **Cell line, EV size, EV concentration, and number of EVs detected with FLIM within 16 fields-of-view.**

Cell Line	Mean Diameter ± SE (nm)		Concentration ± SE (10^7 particles/mL)		Number of EVs detected using FLIM	
	SEVs	LEVs	SEVs	LEVs	SEVs	LEVs
MDA-MB-231	181.1 ± 1.6	214.3 ± 4.9	6.36 ± 0.135	2.38 ± 0.135	132	198
MDA-MB-231	189.8 ± 2.4	214.1 ± 3.9	3.18 ± 0.193	2.34 ± 0.150	176	216
MDA-MB-231	195.7 ± 2.3	209.2 ± 4.5	3.76 ± 0.099	1.74 ± 0.054	150	147

Size and concentration were determined with NTA and concentration is calculated to correspond to EVs/mL of cell culture fluid in T175 cell culture flasks with 30 mL of media per flask.

Supplementary Note 5: EV characterization and validation

For validation, EV samples were imaged with TEM (Phillips CM200, FEI Company, Thermo Fisher Scientific, Hillsboro, OR) to examine their morphology. The initial EV sample after isolation was diluted further in PBS in a 1:99 ratio for TEM. Carbon-coated copper grids were placed in the EV and PBS solution for 3 minutes, then gently dabbed to dry on sterile filter paper. Next, the grids were negatively stained with 2% uranyl acetate for 30 seconds, after which the excess uranyl acetate solution was removed. Grids were then left to dry for 10-15 minutes prior to imaging. Transmission electron microscopy images (**Supplementary Fig. S5a**) showed the expected cup-like morphology and lipid-bilayer membrane enclosed spheres[16,17]. As described in the main text, NTA was also used to characterize samples. NTA size distribution consistently showed particles ranging from 50 to 600 nm with most of the particles concentrated in the 100 to 300 nm range; an example histogram of estimated particle sizes is shown in **Supplementary Fig. S5b**.

EVs were measured with Raman spectroscopy (LabRAM HR 3D, Horiba Scientific, Edison, NJ) for biochemical characterization. Three samples of 10 µL each were dried on coverslips overnight and imaged the following day. Spectroscopy was performed with 532 nm laser excitation, filter optical density of 0.3, 100 µm slit, 300 µm confocal aperture, and grating centered at 2000 cm^{-1} with 300 g/mm. Measurements were averaged over 10s with 0.1 s acquisition time. Analysis was performed using Matlab 2018 (Mathworks, Natick, MA). Previous studies using Raman spectroscopy have not examined EVs from MDA-MB-231 cell culture, but many spectral features of the presented data, plotted with Matlab 2018 (Mathworks, Natick, MA), match with the biochemical profiles found in previous studies using EVs derived from other cells and tissues (**Supplementary Fig. S5c**)[18-21]. NTA concentration and size results for all reported experiments are provided in **Supplementary Table S6**.

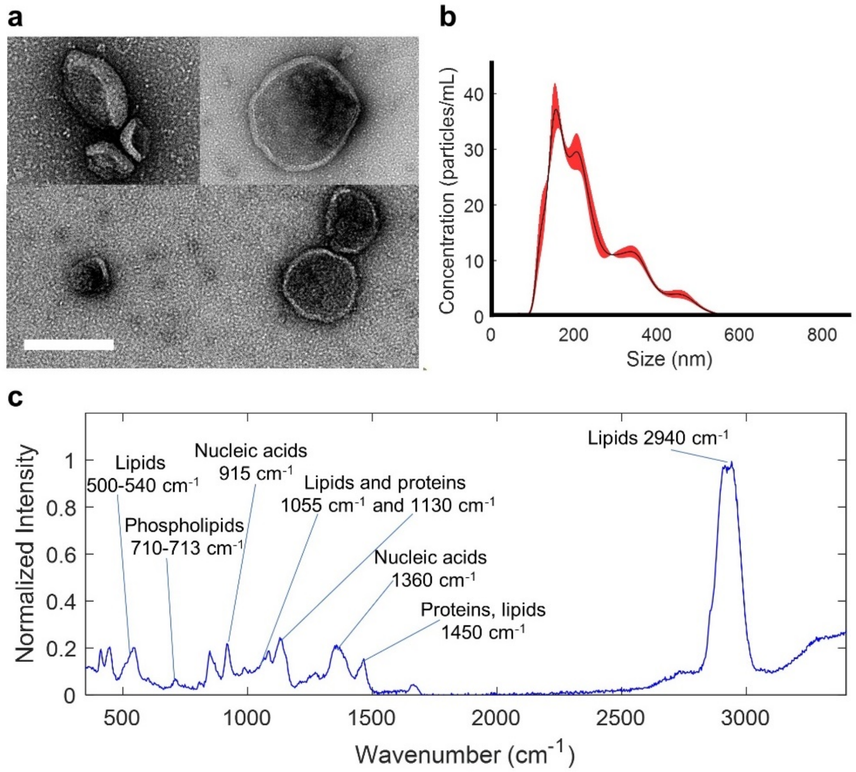

Supplementary Figure S5. **Morphological and biochemical characterization of extracellular vesicles (EVs).** (a) Transmission electron microscopy (TEM) of multiple EV samples show cup-like and more open morphology. (b) Representative concentration density of particles by size determined by nanoparticle tracking analysis (NTA), given in concentration of EVs per mL of cell culture media. (c) Raman spectroscopy data of an EV sample dried on a coverslip overnight. Common peaks are labelled based on previous Raman characterizations of EVs[18-21]. Scale bar represents 200 nm.

Supplementary Table S6. **Cell line, conditions, viability, and concentration for reported experiments.**

Cell Line	Replicate #	Experimental Conditions	Mean Diameter ± SE (nm)	Concentration ± SE (10^7 particles/mL)
MDA-MB-231	1	(**Figure 3a**, **Figure 4a,b**)	214.3 ± 4.9	2.38 ± 0.14
MDA-MB-231	2	(**Figure 3b**, **Figure 4a,b**)	214.1 ± 3.9	2.34 ± 0.15
MDA-MB-231	3	(**Figure 3c**, **Figure 4a,b**)	209.2 ± 4.5	1.74 ± 0.05
J774A.1	1	(**Figure 4c,d**)	199.0 ±1.9	7.25 ± 0.13
J774A.1	2	(**Figure 4c,d**)	199.3 ± 3.2	8.28 ± 0.27
J774A.1	3	(**Figure 4c,d**)	185.4 ± 2.7	8.63 ± 0.43
U87 MG	1	(**Figure 4e,f**)	182.9 ± 5.5	0.87 ± 0.06
U87 MG	2	(**Figure 4e,f**)	184.3 ± 2.4	1.09 ± 0.02
U87 MG	3	(**Figure 4e,f**)	180.6 ± 3.4	1.42 ± 0.07
MDA-MB-231	1	25 mM Glucose (**Figure 6**)	207.3 ± 3.0	12.37 ± 0.41
MDA-MB-231	2	25 mM Glucose (**Figure 6**)	208.6 ± 3.2	12.25 ± 0.43
MDA-MB-231	3	25 mM Glucose (**Figure 6**)	220.9 ± 2.5	15.47 ± 0.37
MDA-MB-231	1	0 mM Glucose (**Figure 6**)	227.9 ± 3.0	21.20 ± 0.51
MDA-MB-231	2	0 mM Glucose (**Figure 6**)	240.7 ± 2.6	19.07 ± 0.43
MDA-MB-231	3	0 mM Glucose (**Figure 6**)	239.0 ± 2.8	29.33 ± 0.70
MDA-MB-231	1	5 mM pyruvate (**Figure 6**)	224.7 ± 2.2	1507 ± 0.46
MDA-MB-231	2	5 mM pyruvate (**Figure 6**)	218.9 ± 2.2	14.40 ± 0.41
MDA-MB-231	3	5 mM pyruvate (**Figure 6**)	231.0 ± 2.2	18.80 ± 0.27
MDA-MB-231	1	5 mM lactate (**Figure 6**)	227.0 ± 3.9	19.60 ± 0.45
MDA-MB-231	2	5 mM lactate (**Figure 6**)	228.3 ± 2.0	21.87 ± 0.50
MDA-MB-231	3	5 mM lactate (**Figure 6**)	238.4 ± 1.3	23.60 ± 0.37
MDA-MB-231	1	100 μM $CoCl_2$ (**Figure 6**)*		
MDA-MB-231	2	100 μM $CoCl_2$ (**Figure 6**)	213.7 ± 3.2	12.31 ± 0.24
MDA-MB-231	3	100 μM $CoCl_2$ (**Figure 6**)	227.1 ± 0.8	18.13 ± 0.17

Concentration is calculated to correspond to EVs/mL of cell culture fluid in T175 cell culture flasks with 30 mL of media per flask. * indicates sample was not measured.

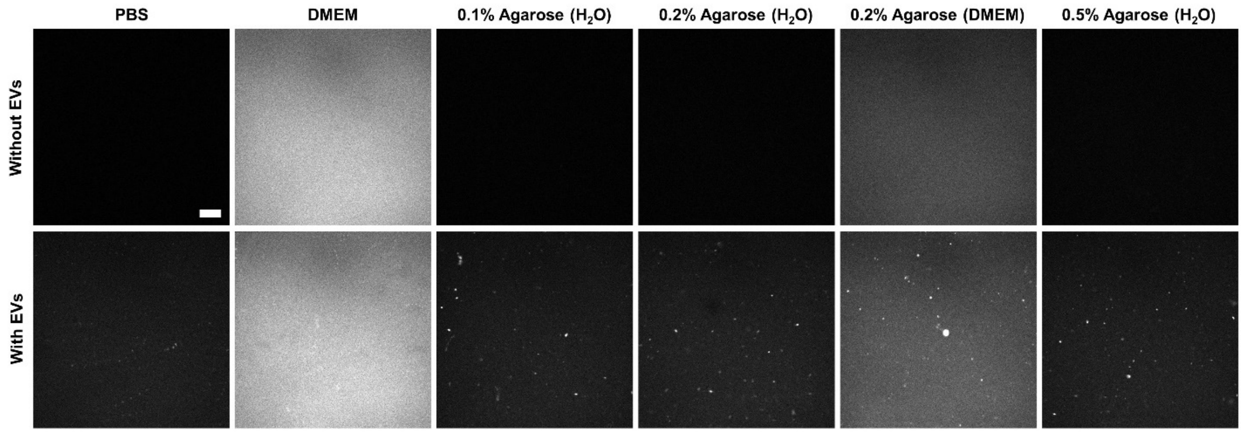

Supplementary Figure S6. **NAD(P)H autofluorescence intensity of extracellular vesicles (EVs) in different solutions.** Solutions with and without MDA-MB-231 derived EVs were imaged under the same conditions as described in the main text. Samples included: phosphate buffer solution (PBS), fetal bovine serum-free Dulbecco's Modified Eagle Medium (DMEM), agarose gels of 0.1% (w/vol), 0.2% (w/vol), and 0.5% (w/vol) made with water, and one agarose gel of 0.2% (w/vol) made with DMEM instead of water. All images are displayed with the same intensity scale. Scale bar represents 20 μm.

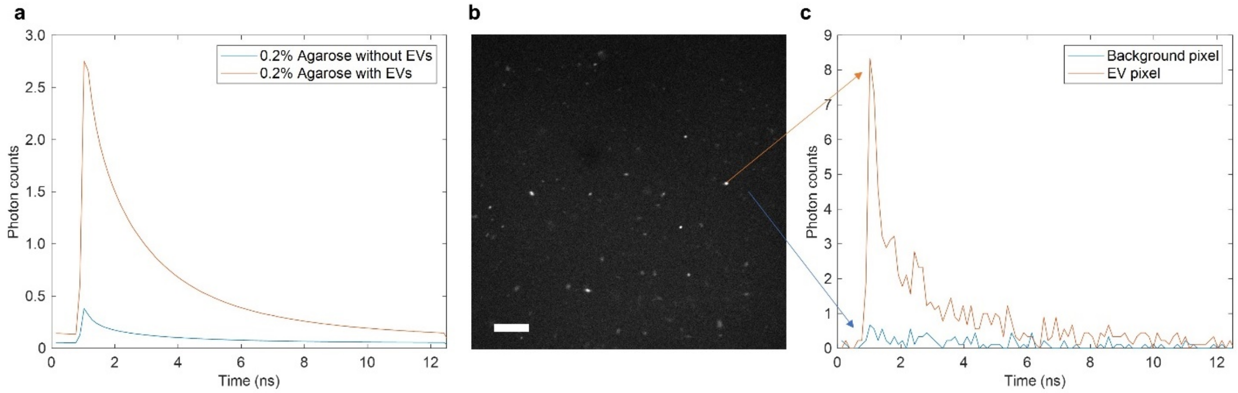

Supplementary Figure S7. **Comparison of autofluorescence signal and background of extracellular vesicles (EVs) in 0.2% (w/v) agarose gel.** (a) Photon count histograms made from the sum of all pixels from 16 fields-of-view (FOV) from 0.2% (w/v) agarose gel made with water without (*blue*) and with (*orange*) MDA-MB-231 derived EVs. (b) Example FOV of MDA-MB-231 derived EVs in a 0.2% (w/v) agarose gel made with water. (c) Photon count histogram from nearby single pixels within the image in (b) without (*blue*) and with (*orange*) MDA-MB-231 derived EV signal. Scale bar represents 20 μm.

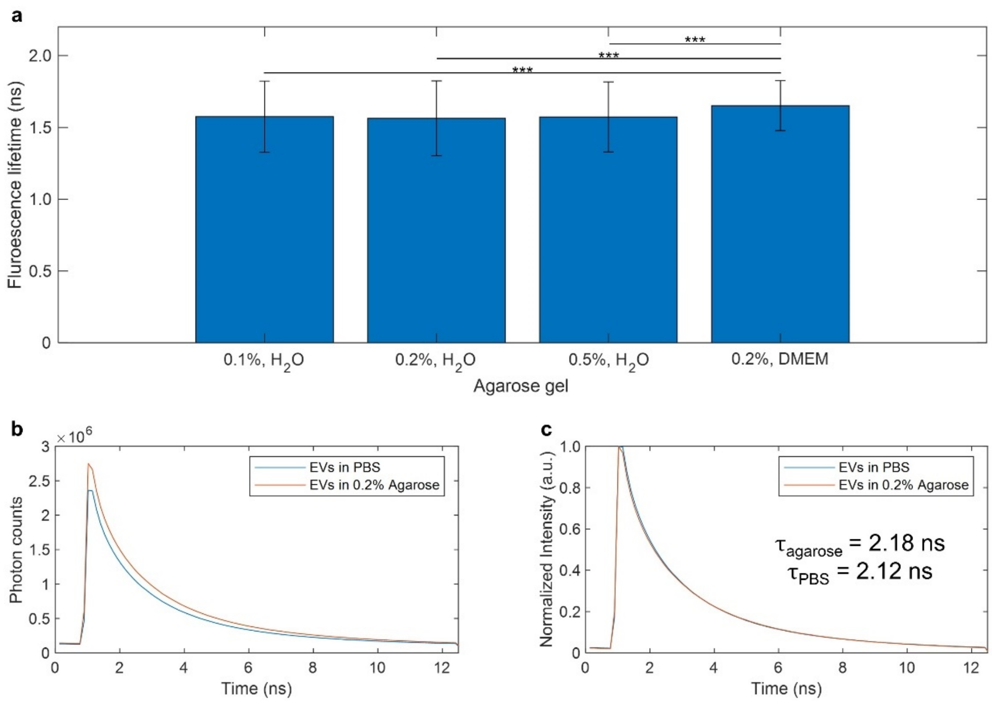

Supplementary Figure S8. **Comparison of extracellular vesicle (EV) NAD(P)H fluorescence lifetime in different solutions.** (a) Fluorescence lifetime of MDA-MB-231 derived EVs in agarose gels of 0.1% (w/vol), 0.2% (w/vol), and 0.5% (w/vol) made with water, and one agarose gel of 0.2% (w/vol) made with DMEM instead of water. No significant difference were found between the three samples made with water. All three samples were significantly different ($P < 0.001$) than the sample made with DMEM, likely due to the high background contribution, and can be seen in **Supplementary Fig. S6**. (b) Photon count histograms made from the sum of all pixels from 16 fields-of-view (FOV) from MDA-MB-231 derived EVs in PBS and 0.2% agarose gel made with water. (c) Normalized photon count histograms from panel (b). The signal in panels (b) and (c) were calculated without EV segmentation and contain contribution from the background as well as the signal, which contributes to the higher fluorescence lifetime.

REFERENCES

1. Liao, S.-C., Sun, Y., Coskun. Y. FLIM analysis using the phasor plots. *ISS Technical Note* (2014).
2. Berezin, M.Y., Achilefu, S. Fluorescence lifetime measurements and biological imaging. *Chem Rev.* **110**: 2641-2684 (2010).
3. Suhling, K., French, P.M.W., Phillips, D. Time-resolved fluorescence microscopy. *Photochem & Photobiol Sci.* **4**:13-22 (2004).
4. Digman, M.A., Caiolfa, V.R., Zamai, M. & Gratton, E. The phasor approach to fluorescence lifetime imaging analysis. *Biophys. J.* **94**, L14-L16 (2008).
5. Mears, R. et al. Proteomic analysis of melanoma-derived exosomes by two-dimensional polyacrylamide gel electrophoresis and mass spectrometry. *Proteomics* **4**, 1019-4031 (2004).
6. Bodega, G. et al. Young and especially senescent endothelial microvesicles produce NADPH: The fuel for their antioxidant machinery. *Ox. Med. Cell. Longev.*, 3183794 (2018).
7. Choi, D. et al. Quantitative proteomic analysis of trypsin-treated extracellular vesicles to identify the real-vesicular proteins. *J. Extracell Vesicles* **9**, 1757209 (2020).
8. Guha, D. et al. Proteomic analysis of cerebrospinal fluid extracellular vesicles reveals synaptic injury, inflammation, and stress response markers in HIV patients with cognitive impairment. *J. Neuroinflammation* **16**, 254 (2019).
9. Wang, S., Kojima, K., Mobley, J.A. & West, A.B. Proteomic analysis of urinary extracellular vesicles reveal biomarkers for neurologic disease. *EBioMedicine* **45**, 351-361 (2019).
10. Melo, S.A. et al. Glypican1 identifies cancer exosomes and facilitates early detection of cancer. *Nature* **523**, 177-182 (2015).
11. Conde-Vancells, J. et al. Characterization and comprehensive proteome profiling of exosomes secreted by hepatocytes. *J. Proteome Res.* **7**, 5157-5166 (2008).
12. Fevrier, B. et al. Cells release prions in association with exosomes. *Proc. Natl. Acad. Sci.* **101**, 9683-9688 (2004).
13. Hegmans, J.P. et al. Proteomic analysis of exosomes secreted by human mesothelioma cells. *Am. J. Pathol.* **164**, 1807-1815 (2004).
14. Zumaquero, E. et al. Exosomes from human lymphoblastoid B cells express enzymatically active CD38 that is associated with signaling complexes CD81, Hsc-70, and Lyn. *Exp. Cell. Res.* **316**, 2692-2706 (2010).
15. Morandi, F. et al. Microvesicles released from multiple myeloma cells are equipped with ectoenzymes belonging to canonical and non-canonical adenosinergic pathways and produce adenosine from ATP and NAD+. *Oncoimmunology* **7**, e1458809 (2018).
16. Van der Pol, E., Boing, A.N., Harrison, P., Sturk, A. & Nieuwland, R. Classification, functions, and clinical relevance of extracellular vesicles. *Pharmacol. Rev.* **64**, 676-705 (2012).
17. Ettelaie, C., Collier, M.E., Maraveyas, A., Ettelaie, R. Characterization of physical properties of tissue factor-containing microvesicles and a comparison of ultracentrifuge-based recovery procedures. *J. Extracell. Vesicles* **3**, 23592 (2014).
18. Gualerzi, A. et al. Raman spectroscopy as a quick tool to assess purity of extracellular vesicle preparation and predict their functionality. *J. Extracell. Vesicles* **8**, 1568780, 2019.
19. Gualerzi, A. et al. Raman spectroscopy uncovers biochemical tissue-related features of extracellular vesicles from mesenchymal stromal cells. *Sci. Rep.* **7**, 9820 (2017).
20. Enciso-Martinez, A. et al. Label-free identification and chemical characterization of single extracellular vesicles and lipoproteins by synchronous Rayleigh and Raman scattering. *J. Extracell. Vesicles* **9**, 1730134 (2020).

21. Tatischeff, I., Larguet, E., Falcon-Perez, J.M., Turpin, P.Y. & Kruglik, S.G. Fast characterization of cell-derived extracellular vesicles by nanoparticles tracking analysis, cryo-electron microscopy, and Raman tweezers microscopy. *J. Extracell. Vesicles* **1**, 19179 (2012).
